# Isolation of anticancer bioactive secondary metabolites from the sponge-derived endophytic fungi *Penicillium sp*. and *in-silico* computational docking approach

**DOI:** 10.3389/fmicb.2023.1216928

**Published:** 2023-10-02

**Authors:** Kumaravel Kaliaperumal, Limbadri Salendra, Yonghong Liu, Zhiran Ju, Sunil Kumar Sahu, Sanniyasi Elumalai, Kumaran Subramanian, Nahaa M. Alotaibi, Nawaf Alshammari, Mohd Saeed, Rohini Karunakaran

**Affiliations:** ^1^Unit of Biomaterials Division, Department of Orthodontics, Saveetha Dental College and Hospitals, SIMATS, Saveetha University, Chennai, India; ^2^New Use Agriculture and Natural Plant Products Program, Department of Plant Biology, Rutgers University, New Brunswick, NJ, United States; ^3^Key Laboratory of Tropical Marine Bio-Resources and Ecology, Center for Marine Microbes, South China Sea Institute of Oceanology, Chinese Academy of Sciences, Guangzhou, China; ^4^Institute of Pharmaceutical Science and Technology, Zhejiang University of Technology, Hangzhou, China; ^5^State Key Laboratory of Biocontrol, Guangdong Provincial Key Laboratory of Plant Resources, Sun Yat-sen University, Guangzhou, China; ^6^Department of Biotechnology, University of Madras, Guindy Campus, Chennai, Tamil Nadu, India; ^7^Research Department of Microbiology, Sri Sankara Arts and Science College (Autonomous), Kanchipuram, Tamil Nadu, India; ^8^Department of Biology, College of Science, Princess Nourah bint Abdulrahman University, Riyadh, Saudi Arabia; ^9^Department of Biology, College of Science, University of Hail, Hail, Saudi Arabia; ^10^Unit of Biochemistry, Faculty of Medicine, AIMST University, Semeling, Bedong, Malaysia; ^11^Centre for Excellence for Biomaterials Science AIMST University, Semeling, Bedong, Malaysia; ^12^Department of Bioinformatics, Saveetha School of Engineering, Saveetha University, Chennai, India

**Keywords:** anticancer, averufin, fungi, HL60, molecular docking, sponges, cytotoxicity, environment

## Abstract

**Introduction:**

Fungus-derived secondary metabolites are fascinating with biomedical potential and chemical diversity. Mining endophytic fungi for drug candidates is an ongoing process in the field of drug discovery and medicinal chemistry. Endophytic fungal symbionts from terrestrial plants, marine flora, and fauna tend to produce interesting types of secondary metabolites with biomedical importance of anticancer, antiviral, and anti-tuberculosis properties.

**Methods:**

An organic ethyl acetate extract of *Penicillium verruculosum* sponge-derived endophytic fungi from *Spongia officinalis* yielded seven different secondary metabolites which are purified through HPLC. The isolated compounds are of averufin (1), aspergilol-A (2), sulochrin (3), monomethyl sulochrin (4), methyl emodin (5), citreorosein (6), and diorcinol (7). All the seven isolated compounds were characterized by high-resolution NMR spectral studies. All isolated compounds', such as anticancer, antimicrobial, anti-tuberculosis, and antiviral, were subjected to bioactivity screening.

**Results:**

Out of seven tested compounds, compound (1) exhibits strong anticancer activity toward myeloid leukemia. HL60 cell lines have an IC_50_ concentration of 1.00μm, which is nearly significant to that of the standard anticancer drug taxol. A virtual computational molecular docking approach of averufin with HL60 antigens revealed that averufin binds strongly with the protein target alpha, beta-tubulin (1JFF), with a −10.98 binding score. Consecutive OSIRIS and Lipinski ADME pharmacokinetic validation of averufin with HL60 antigens revealed that averufin has good pharmacokinetic properties such as drug score, solubility, and mutagenic nature. Furthermore, aspergilol-A (2) is the first report on the *Penicillium verruculosum* fungal strain.

**Discussion:**

We concluded that averufin (1) isolated from *Penicillium verruculosum* can be taken for further preliminary clinical trials like animal model *in-vivo* studies and pharmacodynamic studies. A future prospect of *in-vivo* anticancer screening of averufin can be validated through the present experimental findings.

## 1. Introduction

Marine natural products are of great interest in the field of the pharmaceutical industry, where diverse classes of bioactive substances are derived from marine organisms. Marine sponges are sessile invertebrates that contribute the majority of natural products that possess antiviral, anticancer, antiprotozoal, antifungal, and anti-inflammatory properties (Mehbub et al., 2014). Sponges produce these bioactives as a chemical defense to protect them from predators (Pawlik et al., 2002). Marine sponges are a rich source of natural products that exhibit a wide range of chemical diversity, including xanthones, alkaloids, steroids, cyclic peptides, isoprenoids, quinones, and terpenes. Whether these natural products are secreted independently or by their endophytic symbionts has long been a contentious topic. Endophytic fungi derived from marine and terrestrial fauna and flora possess immense biomedical values in terms of therapeutic targets. Endophytic fungi, *Pullularia* sp., isolated from a terrestrial plant *Culophyllum* sp., produces cytotoxic hexadepsipeptides named pullulans A-D, which exhibited cytotoxicity against human epidermoid cancer cells and human breast cancer cells. (Thomas Edison et al., 2020). Species richness in fungal endophytic diversity was quantified from a Chinese medicinal plant (Cycdales) *Cycas debaoensis* and *Cycas fairylakea*, revealing that these plants possess 33 known genera and 62 different species of fungal endophytes, which include *Taloromyces, Penicillium*, and *Fusarium* spp. (Pecundo et al., 2021). Marine-derived endophytic fungi yielded many bioactive compounds. One such type is Malformin A1, an anti-trypanosomal cyclic peptide that was isolated from a marine seaweed-derived fungi *Aspergillus tubingensis*, followed by isolation of 5-hydroxy-2-pyrone derivatives isolated from green algae *Enteromorpha tubulosa* to produce cAMP on GPR12 cells (Notarte et al., 2017, 2018). Mangroves, a marine plant, harbor enormous endosymbiotic fungi, and various reports share that mangrove-derived endophytic fungi possess antimicrobial properties (Ramirez and Notarte, 2020).

Marine-derived fungi tend to be a potential candidate for bioactive compounds. Fungi associated with those marine organisms, majorly as invertebrates such as sponges, mangroves, and marine algae, were found to represent a vast untapped reservoir of metabolic diversity with growing attention during recent years (Suay et al., 2000; Bugni and Ireland, 2004; Paz et al., 2010; Rateb and Ebel, 2011). These secondary metabolites are products synthesized by the symbiotic-associated microbes within the sponges, such as cyanobacteria, microalgae, fungi, archaea, and bacteria (Unson et al., 1994; Koopmans et al., 2009). Therefore, much attention has been paid to these endophytic microbes and their cultural condition. Since harvesting sponges for their immense pharmacological importance may endanger their community, an effective and alternate method of trapping such endophytes and culturing them in laboratory conditions may yield the targeted secondary metabolites on a pilot scale.

*Spongia officinalis* is a marine sponge that has been used for bioprospecting for many years. An interesting class of chemical moieties like furanosesterpenes and scalarene sesquiterpenes with antibacterial and anticancer properties were isolated from *Spongia officinalis* (Manzo et al., 2011). Whether these bioactive compounds are produced by host organisms or from their endophytic symbionts is a topic of debate. As an ongoing attempt of our continuous exploration for bioactive compounds from the sponge-derived endophytes (Yang et al., 2009; Sun et al., 2015; Wang et al., 2015a), we isolated a fungal strain *Penicillium verruculosum* (XWSO1F60) derived from a marine sponge *Spongia officinalis*. The fungus was fermented on a large scale and extracted, and its bioactive compound isolation was conducted through chromatographic purification, like HPLC. Structural elucidation of isolated compounds was carried out through NMR spectroscopic studies. Herein, we have reported on the fermentation, extraction, and isolation strategies of those bioactive metabolites.

## 2. Materials and methods

### 2.1. General experimental procedures

NMR studies were recorded on a Bruker AC 500MHz NMR (Bruker, Fällanden, Switzerland) spectrometer using TMS as an internal standard. Chemical shifts were expressed in “d” (ppm) and coupling constant ‘J' in Hz. HR-ESI-MS were measured using a Bruker micro TOF-QII mass spectrometer (Bruker, Fällanden, Switzerland). Size exclusion chromatography was conducted using Sephadex LH-20 gel (GE Healthcare, Uppsala, Sweden). Column chromatography was performed using a silica gel (200–300) at Qingdao Marine Chemical Factory (Qingdao, China). TLC spots were detected under UV light and by heating after spraying with 12% H_2_SO4 in H_2_O. Semi-preparative HPLC (RP-HPLC) was conducted on Agilent HPLC (Agilent 1260 infinity series with DAD detector, Santa Clara, CA, USA). All the positive control standard drugs used in the bioassay were procured from Sigma Aldrich (USA).

### 2.2. Sponge material collection

Fresh sponges of *S. officinalis* were collected from the Xidao Island (18^o^23.18'N and 109^o^36.71'E), Hainan province of the South China Sea coast, in July 2014, during a marine cruise collection. Sponges were transported to the laboratory in ice-freeze conditions and stored at−20^o^C until further use. Sponges were identified based on morphological and spicule identification by the Department of Marine Bioresources and Ecological Sciences, SCISO, China. A voucher specimen (Voucher Number: SCISO 45874/2018) was deposited at SCSIO marine biological collections.

### 2.3. DNA extraction and phylogenetic sequence analysis

The endophytic fungus XWSO1F60 was isolated from the sponge *S. officinalis*. The strain was grown on a MactoBalt (MB) agar slant at 25°C. Two-week-old fungal hyphae were scraped for genomic DNA isolation using the Ultraclean Microbial DNA Kit (MoBio Laboratories). The internal transcribed spacer of ribosomal nucleotide sequence (ITS rDNA) primers ITS1 (5′-GCACAGGCAGCAGGAGCTG CCCCTCAGCTGTCTCCTCGTGCTCAAC-3′) and ITS (5′- AGAGCAAGCCGAGCAGGTCTATCGCCAAGTATCCTCAG AAGCTGTGCT-3′) were used (Volkov et al., 2014). The PCR reaction was conducted using Eppendorf equipment (Eppendorf, NY, USA). The reaction mixture of 50 μL consisted of polymerase chain reaction (PCR) buffer, 2.5 mM Mg^2+^, 100 μM dNTPs, 0.5 μM each primer, 10 ng extracted DNA, and 2 U Taq polymerase. The thermocycling steps involved an initial denaturation at 95^o^C for 5 min, followed by 20 cycles consisting of 1.5 min at 95^o^C, 2 min at 50^o^C, and 2 min at 68^o^C. This was followed by another set of 20 cycles with 10 min at 68 C and a final extension step of 10 min at 4^o^C. The resulting PCR product was processed from agarose gel using QIA quick Gel Extraction Kit (QIAGEN, Valencia, CA, USA) and sequenced using an ABI 3730 sequencer (Applied Bio-systems, USA). Sequences were analyzed using the BLAST program (Basic Local Alignment Search Tool). The phylogenetic tree was constructed based on the neighbor-joining (NJ) method using MEGA-5.0 by using 1,000 bootstrap replicates. The sequence was deposited in GenBank and assigned an accession number (Genbank: KU891245).

### 2.4. Fermentation, extraction, and isolation of compounds

A stock culture of the strain XWSO1F60 was grown on MB agar solid medium at 25^0^C for a week. The stock culture was inoculated in an optimized seed medium (malt extract 15 g, sea salt 2.5 g, NaCl 2.5 g, distilled water 1000 mL, pH 7.4 −7.8) and incubated at 25^0^C for 72 h on a rotating shaker (180 rpm). The optimization of the growth medium was selected based on previous experimental validation with maximum fungal growth and UV-HPLC metabolite fingerprinting. The mass quantity of fermentation of fungal isolates XWSO1F60 was carried out using a solid rice medium in 1000 mL flasks (rice 200 g, sea salt 2.5 g, distilled water 200 mL), which were inoculated with 10 mL of seed solution. Flasks were incubated at 25^o^C under a day-night cycle. 60 days old of fungal cultures from 40 flasks were subjected to organic extraction using Acetone /Ethyl acetate (EtOAc). The combined acetone/EtOAc fungal culture extracts were partitioned between 90% aqueous MeOH and petroleum ether. The resultant MeOH yield was fractionated using a silica column, Sephadex LH-20, and finally, semi-preparative reversed-phase HPLC to yield compounds (1–7).

The EtOAc crude extracts were then purified by silica gel column chromatography eluted with CHCL_3_-MeOH in a gradient eluent (v/v, 75:1, 50:1, 30:1, 20:1, 5:1, 1:1, 0:1) to obtain fractions 1–8 based on the TLC spots. Fr. 4 (950 mg) was purified by Sephadex LH-20 (CHCl_3_/MeOH, 1:1) to obtain three subfractions (fr. 4.1–4.3). Fr.4.1 (226 mg) was further purified by SP-RP HPLC eluting with CH_3_CN/H_2_O (55:45) to afford compound diorcinol (7) (16.7 mg) and compound methyl emodin (5) (9.6 mg). Fraction 3 (1.9 g) was purified using Sephadex LH-20 (CHCl_3_/MeOH, 1:1) and SP-RP HPLC using a C-18 column (Agilent 1260 infinity, YMC-Pack, ODS-A S-5 μm × 12 nm 250 × 20mm i.d., 3 mL per minute) eluting with MeOH/H_2_O (10:90) to afford compound citreoresin (6) (13.2 mg). Fr. 7 (1.8 g) was subjected to Silica gel chromatography eluted with Acetone/MeOH in a step-wise eluent (1:8, 3:5, 3:3, 5;1, 8:1) to give three subfractions (fr. 7.1–7.3). Fr. 7.2 (159 mg) was purified using SP-RP HPLC eluting with CH_3_CN / H_2_O (79:21) to afford compound averufin (1) (5.6 mg) and compound apergilol-A (2) (12.2 mg). Fr. 8 (3.9 g) was subjected to silica gel column chromatography eluted with a CHCL_3_/MeOH in a gradient elution of (50:1, 35:1, 15:1, 10:1 and 0:100) (v/v), which yielded six fractions (fr 8.1 – 8.3). Fr. 8.3 (700 mg) was subjected to ODS chromatography eluted with MeOH/H_2_O (linear gradient, 50–100% MeOH) to obtain five subfractions (fr. 8.3.1–8.3.3). Fr. 8.3.1 (96.1mg) was further purified using SP-RP HPLC eluting with CH_3_CN- H_2_O (50:50) to afford the compound monomethyl sulochrin (4) (14.9mg). Fr. 8.2.4 (23 mg) was purified using (SP-RP) HPLC eluting with CH_3_CN-H_2_O (67:33) to afford the compound sulochrin (3) (11.6mg).

### 2.5. NMR spectroscopy characterization

All the isolated compounds were checked for purity using Thin-layer Chromatography (TLC). After ensuring the purity, the compounds were subjected to ^1^H and ^13^C-Nuclear Magnetic Resonance (NMR) spectroscopy (500 MHz, Bruker). Trimethylsilane (TMS) was used as an internal standard. The NMR spectrum was analyzed using MestreNova spectral processing (Version. 14.2.0).

### 2.6. Anticancer screening

Cytotoxicity was assessed using the Cell Counting Kit (CCK-8) (Dojindo, Japan) method adopted by Wang et al. (2015a). Cancer cell lines used in this study included K562, MCF-7, A549, HuH-7, H1975, HeLa, HL7702, DU145, HL60, MOLT-4. Additionally, a normal cell line, MCF10A, was included. All cell lines were procured from Shanghai Cell Bank, Chinese Academy of Sciences. Cells were routinely grown and maintained in RPMI or DMEM media with 10% Fetal Bovine Serum and 1% penicillin/streptomycin. Various concentrations of seven isolated compounds from *Penicillium* sp. were dissolved in 100% DMSO with a maximum concentration of 200 μg/mL and serially diluted to a final 0.1% DMSO concentration to treat cells for 2 h. Taxol was used as a positive control, and DMSO was used as a negative control. Cytotoxicity screening assay experiments were conducted in triplicate to obtain the standard error ± mean value. The percentage of cytotoxicity (IC_50_) alongside the selectivity index was then calculated using the formula:


$$\begin{array}{l} {\text{IC}}_{\text{50}}\text{=}(\text{absorbance of the cell without treatment}\\ \text{ -absorbance of cells with treatment/}\\ \text{ absorbance of the cell without treatment})\times \;\text{100} \end{array}$$

$$\begin{array}{l} \text{Selective index (SI)}=\text{C}{\text{C}}_{50}\text{value for normal cells}/\\ \text{I}{\text{C}}_{50}\text{for cancer cells} \end{array}$$

### 2.7. Anti-tuberculosis assay

The H37Ra strain of *Mycobacterium tuberculosis* (ATCC 25177) purchased from the American Type Culture Collection (ATCC) was used in the anti-TB bioassay. The anti-tuberculosis assay was based on the one used by Wang et al. (2015b). INH (Isoniazid) was used as the positive control, and DMSO was used as a negative control. For the minimum inhibitory concentration (MIC) analysis, 100 μl of *Mycobacterium* suspension was prepared in a 96-well microtiter plate. A total of 10 mL of double serial dilution of various concentrations of 7 isolated compounds (from 0.08 to 20 μg/mL) alongside the positive control isoniazid (1 to 417 μg/mL) was added to the well. The anti-tubercular assay was done in triplicate to obtain the SD± mean value.

### 2.8. Antiviral activity

Influenza A virus strains H1N1 (ATCC, VR-1520) and H3N2 (ATCC, VR-1679) were used in the present study. The antiviral activities against H1N1 and H3N2 were evaluated by the CPE inhibition assay based on the methodology (Fang et al., 2014). The IC_50_ was determined by the concentration required to inhibit the influenza virus yield at 48 h post-infection by 50%. The antiviral assay was conducted in triplicate to obtain the SD± mean value.

### 2.9. Antimicrobial assay

The antimicrobial activities against *Staphylococcus aureus* (CGMCC 1.230) and *Escherichia coli* (CGMCC 1.2385) were evaluated by an agar dilution method based on the study by Wang et al. (2014). Microbial strains used in the present study were procured from the China General Microbiological Culture Collection Center (CGMCC). The isolated compounds were dissolved in dimethyl sulfoxide (DMSO) and added to a 96-well plate in a concentration ranging from 0.3 to 50 μg/mL. Then, malto broth liquid medium with grown bacterial suspension was added to each well, and the cell density was adjusted to ~106 cfu/mL. The plates were kept in the incubator at 37°C for 24 h. The minimum inhibitory concentrations (MIC) were denoted at least at a concentration where no microbial growth could be observed. Ciprofloxacin was used as a positive control, and DMSO was used as a negative control. The antimicrobial assay was done in triplicate to obtain the SD± mean value.

### 2.10. Data analysis

Each experimental data obtained after triplicate assays were computed as a standard error deviation. A *p*-value of ≤ 0.05 was calculated as statistically significant using a one-way ANOVA. The analysis was performed using SPSS statistical package version 19.0.

### 2.11. Molecular docking

Computational docking studies based on the structure-activity relationship obtain a better understanding of drug-target interactions. Based on the biological screening test compound (1), averufin was docked with four major cancer antigens of HL60 cell lines, namely, human serum transferrin, CD-5 antigen, CD-20, and alpha-beta tubulin from zinc-induced sheet based upon the methodology derived by Notarte et al. (2023). The antigenic drug targets were selected based on the previous literature survey and prominent antigens that are over-expressed on myeloid leukemia cancer cells (Taetle et al., 1985; Launder et al., 1996; Shariftabrizi et al., 2012; Lagorce et al., 2015). Taxol was used as a standard reference drug. Three dimensional (3D) structures of those target proteins human serum transferrin (1A8E), CD-5 antigen (2JOP), CD-20 (3BKY) antigen, and the refined structure of alpha-beta tubulin from zinc-induced sheets (1JFF), stabilized with taxol (PDB ID: 1A8E, 2JOP, 3BKY, and 1JFF, respectively) were retrieved from the protein data bank (PDB). The chemical structures of the natural inhibitors (taxol), as well as the averufin (1), were generated from SMILES notation (Simplified Molecular Input Line Entry Specification) using the Chemsketch Software.

### 2.12. Protein-ligand docking

For docking analysis, Argus Lab 4.0.1 software was used, followed in accordance to the methodology described by Duverna et al. (2010). The active sites on the target proteins were obtained from RCSB ligand explorer software. The proteins and ligands were geometrically optimized, and hydrogen bonds were added. The genetic algorithm (GA) was used as the docking engine, and the grid resolution was set to 0.40 A^o^. The calculation type was set to “Dock” mode, whereas “flexible mode” was selected for the ligand. The lowest energy represented the easy binding character of ligands and receptors. Molecular interactions between ligands and target proteins were visualized using Discovery Studio (Ver 3.1) software.

### 2.13. *In-silico* pharmacokinetic ADME predictions

The pharmacokinetic properties of compound (1) averufin were predicted by using the Swiss ADME software (Swiss Institute of Bioinformatics, 2019) based on the methodology derived by Quimque et al. (2021a). Pharmacokinetic ADME predictions were evaluated as Lipinski's “rule of five,” which includes the basic molecular weight, hydrogen bond acceptors and donors, and lipophilicity properties of the drug. The boiled egg prediction for compound (1) averufin was also assessed to check the water solubility. Apart from that, the OSIRIS property explorer program (Thomas Sander, Idorsia Pharmaceuticals Ltd., 2017) was employed for assessing the *in-silico* toxicity prediction to evaluate the mutagenicity, tumorigenicity, irritant effects, and reproductive toxicity efficacy of compound (1), i.e., averufin (de Leon et al., 2021; Quimque et al., 2021b; Brogi et al., 2022).

### 2.14. Molecular dynamic study

A molecular dynamic (MD) analysis was performed using Schrödinger maestro based on the methodology of Wu et al. (2018). Targets and ligands with maximum binding energy and clinical relevance based on docking results were selected and subjected to molecular dynamic drug target binding efficacy. Here, the target human serum transferrin (1A8E) protein was selected to dock with compound (1) averufin based on the good docking score. As a prerequisite for the dynamic studies, the parameters were fixed as the tetrahedron water box with water molecules were used to soak, and the edge of the box was 1 nm. Then, the surface charges of complexes were neutralized by adding 30 Na^+^ and 10 Cl^−^. The energy minimization was conducted and equilibrated by NVT at 300 K and 1 bar for 100 ns and then subjected to a molecular dynamics study using an NPT ensemble. A molecular dynamic (MD) prediction of drug and target was performed at 100 nanoseconds (ns) to assess the bonding stability and displacement of ligand with that of the target.

## 3. Results

### 3.1. Identification of fungi

**Figure 1:**
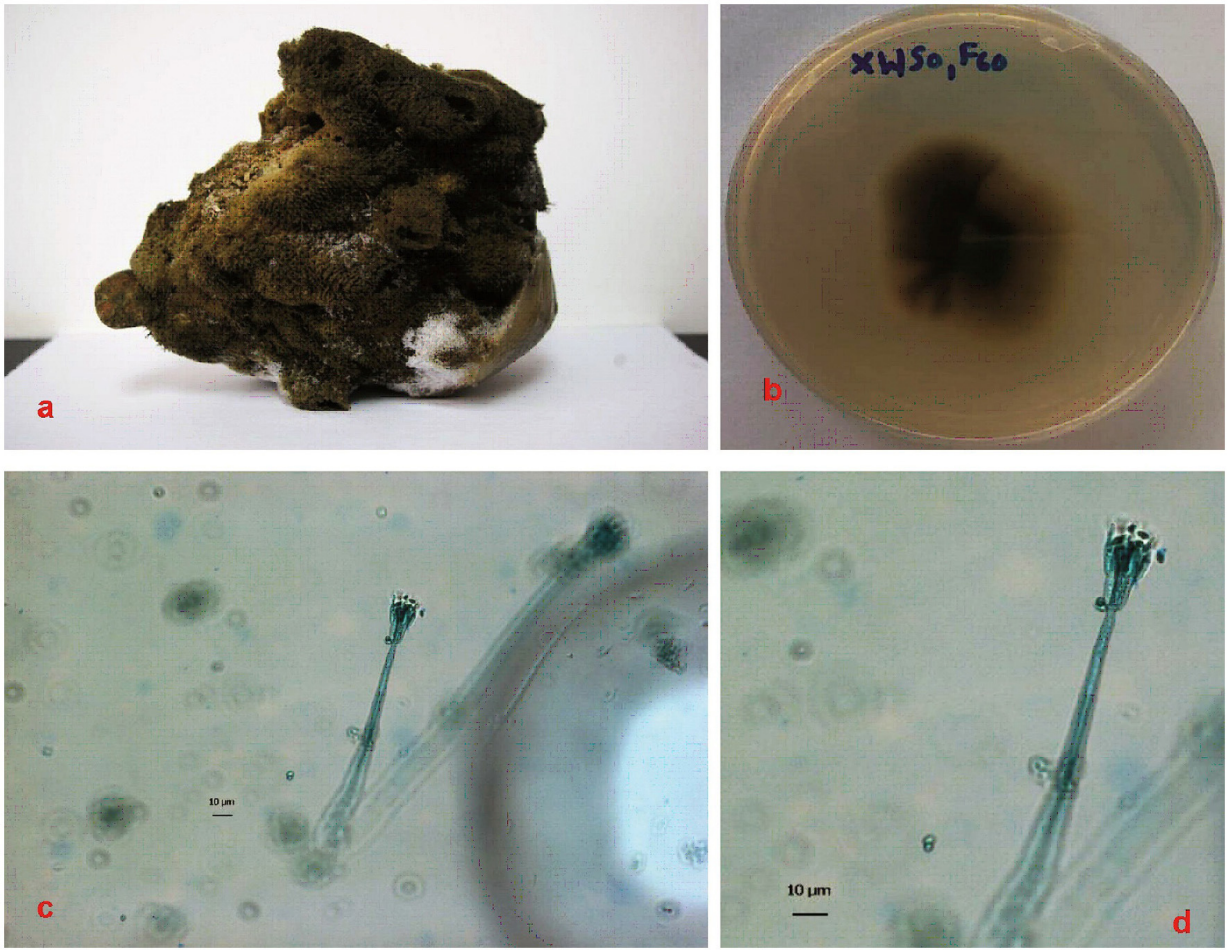
Sponge specimen and fungal colonies. **(a)**: Sponge specimen *S. officinalis*. **(b)**: Fungal colony of *P.verruculosum* (XWSO1 F60) in the MB agar plate. **(c, d)**: Hypha of the fungal colony after 2 weeks under confocal microscopy imaging. Bars in both c and d represent 10 μm.

**Figure 2:**
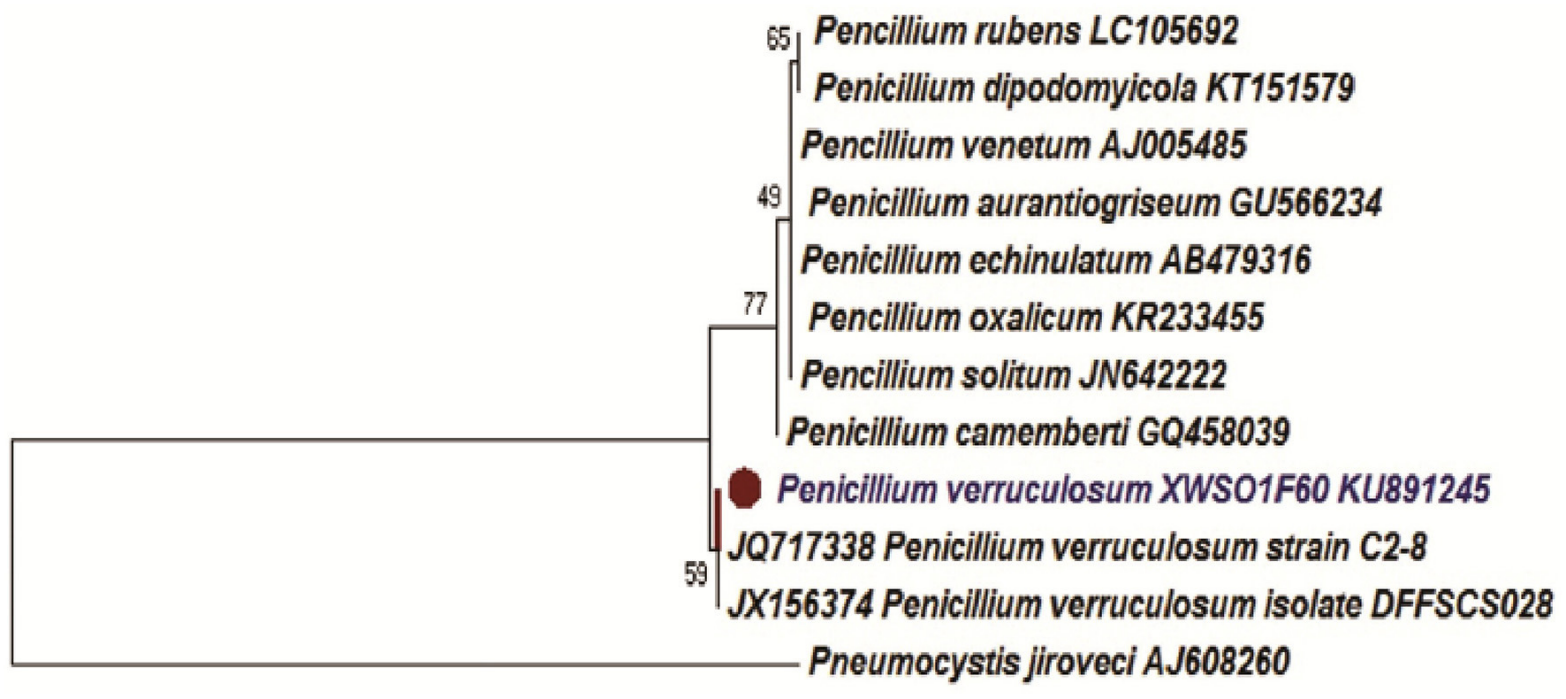
Phylogenetic Neighbor-Joining (NJ) tree inferred from ITS rDNA sequences. Bar represents 0.1 substitutions per site.

Two-week-old fungal colonies reached a diameter of 2–3 cm wide. The colonies appeared pale green by visual observations. Under confocal microscopy (Leica Microsystems, Mannheim, Germany), the fungal hyphae stained with lactophenol blue appears to be slender with dispersed conidiophores (Figure 1). The fungal strain XWSO1F60 was identified as *P. verruculosum* based upon the sequence obtained from the internal transcribed spacer (ITS) regions (Genbank accession number: KU891245), and it has 99% similarity with that of *P.verruculosum* strain C2-8 (JQ717338) (Figure 2).

### 3.2. Metabolite isolation

**Figure 3:**
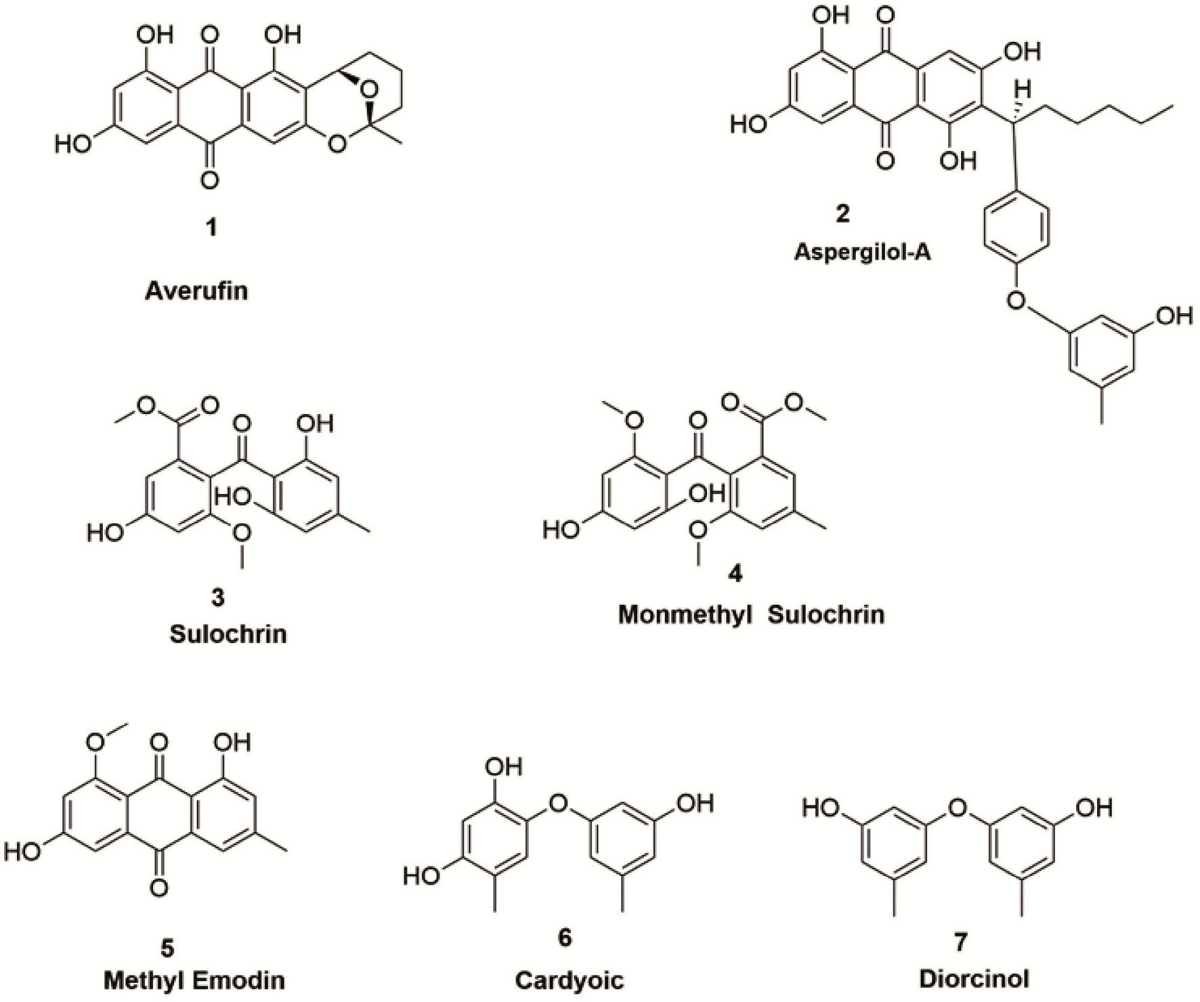
Metabolites isolated from *P.verruculosum* fungi.

The organic extract of *P.verruculosum* strain XWSO1F60 yielded seven different metabolites, which comprise polyketides, xanthones, and alkaloid derivatives. Structural determinations were carried out manually and referred to previously published NMR spectral data for their consistency (Figure 3).

#### 3.2.1. Averufin (1)

Amorphous powder with orange-red color: ^1^H-NMR (500MHz, DMSO): δH: 1.52(3H, s, H-6′ ), 1.62(2H, m, H-4′), 1.72 - 2.01(2H, m, H-3′), 1.82 - 1.99 (2H, m, H-2′), 5.28(1H, d, J = 2 0.5 Hz, H-1′), 6.58(1H, d, J= 2.6 Hz, H-7),7.03(1H, s, H-4), 7.11 (1H, d, J= 2.6 Hz, H-5), 12.109(1H, s, OH-8), 12.54(1H, br s, OH-1); ^13^C-NMR (125 MHz, DMSO): δC: 15.2 (C-4′), 26.8 (C-3′), 27.3 (C-6′), 35.1 (C-2′), 66.1(C-1′), 101.1 (C-5′), 107.1 (C-4), 108.0 (C-7), 108.4 (C-9a), 108.6 (C-8a), 108.9 (C-5), 115.9 (C-2), 133.1 (C-10a), 134.9 (C-4a), 158.1 (C-3), 159.8 (C-8), 164.2 (C-1),165.2 (C-6), 180.9 (C- 10), and 188.8 (C-9) (Hong et al., 2007).

#### 3.2.2. Aspergilol – A (2)

Amorphous Red powder: ^1^H-NMR (500MHz, DMSO): δH: 0.83 (3H, t, J = 6.0Hz, H3-16), 1.22 (1H, m, H-13), 1.26 (2H, m, H-15), 1.26 (2H, m, H-14), 1.40 (1H, m, H-13), 1.75 (1H, m, H-12), 1.87 (3H, s, H-7″), 2.39 (3H, s, H-7′), 2.56 (1H, m, H-12), 4.40 (1H, t, J = 8.0Hz, H-11), 5.66 (1H, br s, H-4″), 5.68 (1H, br s, H-2″), 5.85 (1H, br s, H-6″), 5.96 (1H, d, J = 1.5Hz, H-4′ ), 6.37 (1H, d, J =2.2 Hz, H-2′ ), 6.54 (1H, d, J = 2.2 Hz, H-2), 6.98 (1H, s, H-5), 7.03 (1H, d, J = 2.3 Hz, H-4), 8.98 (s,-OH-5″), 9.14 (br s, - OH-5′), 10.87 (s, OH-3), 11.20 (br s, OH-6), 12.20 (s, OH-1),12.87 (s, OH-8); ^13^C-NMR (125 MHz, DMSO): δC: 14.4 (C-16), 20.7 (C-7′), 21.2 (C-7″), 22.6 (C-15), 28.8 (C-13), 32.1 (C-14), 33.1 (C-12), 37.3 (C-11), 101.5 (C-4″), 105.8 (C-4′), 108.3 (C-2), 108.5 (C-5), 108.5 (C-8a), 108.7 (C-4), 108.8 (C-2″), 109.2 (C-1a), 109.6 (C-6″), 113.7 (C-2′), 122.9 (C-7), 126.4 (C-6′), 131.8 (C-5a), 135.2 (C-4a), 139.1 (C-1″), 139.7 (C-1′), 155.1 (C-5′), 155.9 (C-3′), 158.2 (C-3″), 158.8 (C- 5″), 164.1 (C-3), 164.2 (C-6), 164.4 (C-8), 165.1 (C-1), 181.6 (C-9), and 189.2 (C-10) (Wu et al., 2016).

#### 3.2.3. Sulochrin (3)

Yellow color: ^1^H NMR (500MHz, DMSO): δH: 2.31 (3H, s, CH_3_-7′), 3.64 (3H, s, OCH_3_-3), 3.65 (3H, s, COOCH_3_), 6.09 (1H, s, H-5′ and 3′), 6.68 (1H, d, J = 2.5, H-4), 6.91 (1H, d, J = 2.5, H-6), 10.02 (1H, s, OH-5), 12.48 (1H, s, OH-2′); ^13^C NMR (125 MHz, DMSO): δC: 21.5 (C-7′), 52.1 (C-9′), 55.9 (OCH_3_, C-7), 103.3 (C-4), 107.1(C-6), 107.5 (C-5′ and3′), 109.1 (C-1′), 126.1 (C-2), 127.8 (C-1), 143.3 (C-4′), 156.7 (C-3), 158.1 (C-5), 161.6 (C-6′ and 2′), 165.6 (C-7), and 199.6 (C-8′) (Huang et al., 1996).

#### 3.2.4. Monomethyl sulochrin (4)

Colorless powder: ^1^H NMR (500MHz, DMSO): δH: 2.25 (3H, s, CH_3_-7′), 3.33 (3H, s, OCH_3_-9′), 3.62 (3H, s, OCH_3_-7), 3.63 (3H, s, OCH3-8), 6.26 (1H, s, H-5′), 6.38 (1H, s, H-3′), 6.69 (1H, d, J = 1.5, H-4),6.90 (1H, d, J = 1.5, H-6), 10.19 (1H, s, OH-5), 12.95 (1H, s, OH-2′); ^13^C NMR (125 MHz, DMSO): δC: 21.9 (C-7′), 52.1 (OCH_3_-7), 55.9 (OCH_3_, C-9′),55.9 (OCH_3_-8), 103.1 (C-4),103.5 (C-5′), 107.1 (C-6), 110.1 (C-1′ and 3′), 125.8 (C-2), 127.9 (C-1), 147.8 (C-4′), 156.6 (C-3), 158.1 (C-5), 160.7 (C-6′),163.2 (C-6′),165.7 (C-7), and 199.3(C-8′) (Silva-Silva et al., 2022).

#### 3.2.5. Methyl emodin (5)

Orange powder: ^1^H NMR (500MHz, DMSO):δH: 2.38 (3H, s, H3-3), 3.86 (3H, s, OCH_3_-1), 6.68 (1H, br s, H-7), 7.08 (1H, s, H-2), 7.10 (1H, s, H-5), 7.39 (1H, s, H-4), 13.58 (1H, s, OH-8); ^13^C NMR (125 MHz, DMSO): δC: 21.3 (3-CH_3_), 56.9 (1-OCH_3_), 105.1 (C-7), 108.7 (C-5), 110.6 (C-8a), 114.5 (C-9a), 118.7 (C-4), 123.9 (C-2), 132.1 (C- 10a), 136.6 (C-4a), 145.9 (C-3), 161.6 (C-6), 163.7 (C-1), 167.8 (C-8), 182.8 (C-10), and 185.3 (C-9) (Qian et al., 2011).

#### 3.2.6. Citreorosein (6)

Yellow amorphous solid: ^1^H NMR (500MHz, DMSO):δH: 4.60 (2H, br s, H-6),6.59 (1H, d, J = 2.0, H-2),7.12 (1H, d, J = 2.0, H-4), 7.24 (1H, s, H-7), 7.63 (1H, s, H-5), 12.06 (1H, d, J = 14, OH-1); ^13^C NMR (125 MHz, DMSO): δC: 61.9 (CH_2_OH),107.9 (C-2),108.9 (C-4), 108.9 (C-8a), 114.1 (C-9a), 117.0 (C-7),120.7 (C-5), 132.9 (C-4a), 135.1 (C-10a),152.8 (C-6), 161.5 (C-3), 164.3 (C-8), 165.5 (C-1), 181.3 (C-10), and 189.6 (C-9) (Ren et al., 2006).

#### 3.2.7. Diorcinol (7)

Brown oil: ^1^H NMR (500MHz, CD3OD):δH: 2.23 (6H, s, H3-7 and 7′), 6.23 (2H, br s, H2-2 and 2′), 6.29 (2H, br s, H2-4 and 4′), 6.39 (2H, br s, H2-6, and 6′); ^13^C NMR (125 MHz, CD3OD): δC: 21.5 (CH_3_-5), 104.2 (C-2,), 111.7 (C-4 and C-6), 112.1 (C-4′and C-6′), 141.6 (C-5), 159.5 (C-1 and C-3), and 159.5 (C-1′, and C-3′) (Zhang et al., 2014).

### 3.3. Biological screening

#### 3.3.1. Anticancer assay

Anticancer screening for all the seven compounds assessed using *in-vitro* methods revealed that compounds averufin (1) and methyl emodin (5) exhibit a significant anticancer effect against cancer cell lines compared to the others. Compound (1) shows strong anticancer activity toward HL60 cells with an IC_50_ value of 1.005 μM concentration, and compound (5) shows moderate activity (Table 1). The rest of the compounds did not show any positive anticancer effects (data not shown due to any efficient activity).

**Table 1:** Anticancer activity of compounds from *P. verruculosum*.

**Cpd**	**K562**	**A549**	**DU145**	**H1975**	**MCF-7**	**Huh-7**	**HL7702**	**HL60**	**HeLa**	**MOLT-4**	**MCF-10A**
**1**	17.4 ± 0.01	76.1± 0.01	91.2± 0.01	8.64± 0.01	6.71± 0.01	3.13± 0.01	3.57± 0.01	1.005± 0.0^*^	8.11± 0.01	5.77± 0.01	≥50
**5**	16.3 ± 0.01	81.3± 0.01	101.4± 0.01	43.5± 0.01	54.3± 0.01	25.1± 0.01	67.2± 0.01	13.2± 0.01	24.9± 0.01	13.6± 0.01	≥50
**Taxol**	0.003 ± 0.01	0.024± 0.01	0.015± 0.01	0.014± 0.01	0.002± 0.01^*^	0.003± 0.01	0.003± 0.01	0.002± 0.01^*^	0.003± 0.01	0.003± 0.01	≥50
**SI**	1.22	1.87	3.11	1.24	1.78	2.47	3.89	13.47	3.24	2.47	69.41

Data are computed as standard deviation mean ± SD, n = 3, SI (selective index),

* p ≤ 0.05 (one-way ANOVA).

#### 3.3.2. Antimicrobial, antiviral, and anti-tubercular assays

None of the tested compounds was recorded with any positive antimicrobial, anti-tuberculosis, or antiviral activity in the screening, except for anti-tuberculosis INH (Isoniazid), with a MIC value of 3.98μM, which was recorded. For antiviral, Tamiflu was used as the positive control with IC_50_ values of 15.2 and 17.6nM, respectively, and for antimicrobial, ciprofloxacin was used as the positive control for *S. aureus* and *E. coli* with MIC values of 2.96 and 0.19μM, respectively.

#### 3.3.3. Molecular docking

**Figure 4:**
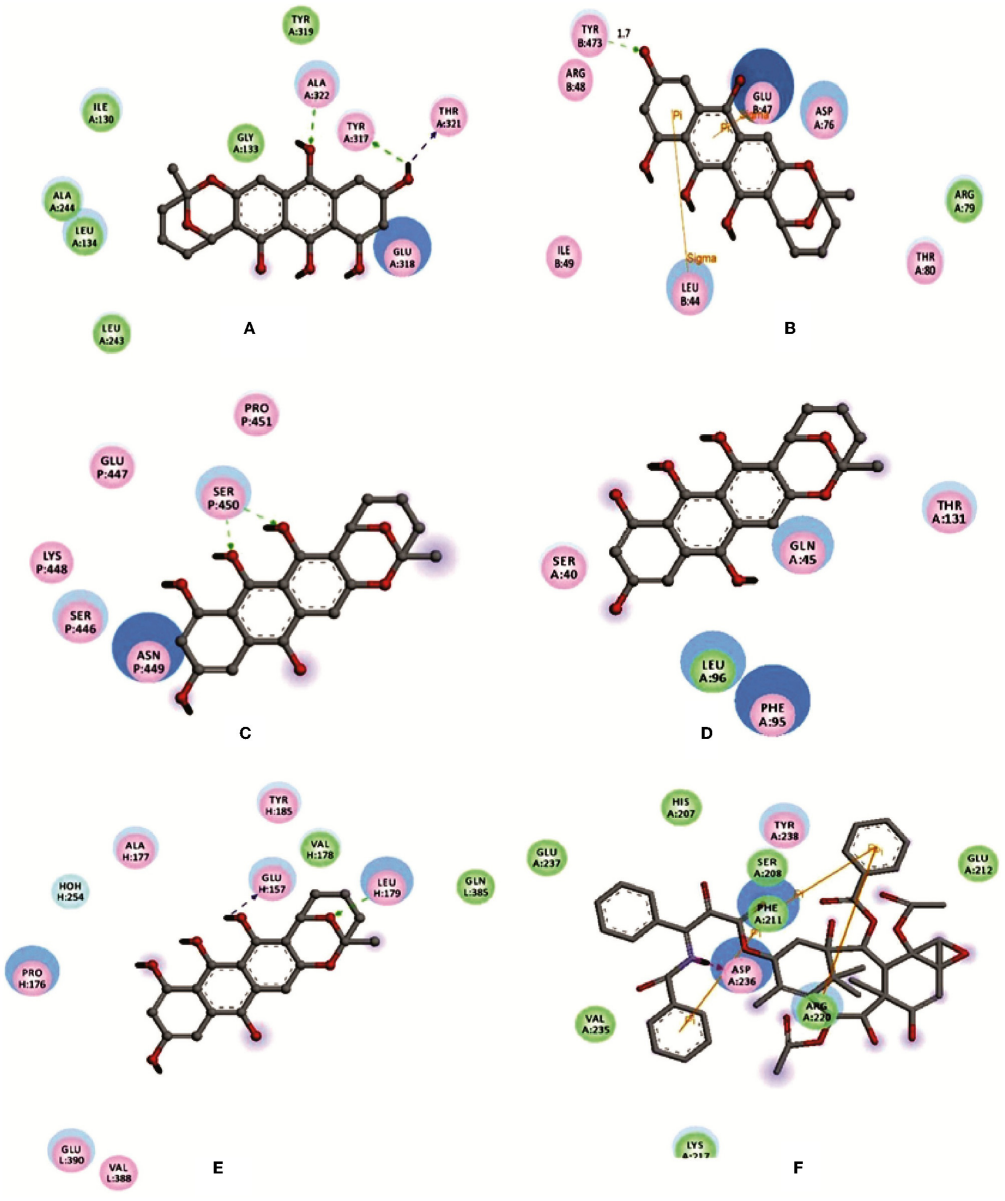
Depicting the molecular visualization of protein-ligand interactions. The interactions were visualized in Discovery Studio software. **(A)**: 1A8E and averufin. **(B)**: 1JFF and averufin. **(C)**: 2JOP and averufin. **(D)**: 3BKY_chain-H and averufin. **(E)**: 3BKY_chain-P and averufin. **(F)**: 1A8E and taxol. The dashed arrow shows the hydrogen bond.

Averufin (1) showed a better docking score when compared to the standard drug Taxol against all the target proteins, as evidenced by the protein-ligand interaction (Figure 4 and Table 2). The interacting amino acids present in the binding site and the hydrogen bonds are shown in Figure 4. The docking score ranged from −6.2627 to −10.2202 Kcal/mol. The best docking score of −9.0467 was observed against the 1A8E ligand, which corresponds to human transferring protein and is highly significant when compared to that of the Taxol drug. Since the protein 1JFF alpha-beta tubulin exerted a higher binding score of −10.2202 Kcal/mol, which was also significantly higher than the standard Taxol (−8.93775 Kcal/mol), the binding energy with 1A8E is very prominent. Similarly, averufin (1) showed quite a better docking hit against other target proteins. The docking study substantiates the *in-vitro* results.

**Table 2:** Molecular docking score against the target proteins.

**Compound**	**1A8E (Kcal/mol)**	**2JOP (Kcal/mol)**	**3BKY (Chain P) (Kcal/mol)**	**3BKY** **(Chain H) (Kcal/mol)**	**1JFF (Kcal/mol)**
Averufin	−9.0467	−8.28216	−6.2627	−7.69887	−10.2202
Taxol	−8.4532	−8.30715	−2.51873	−7.30745	−8.93775

#### 3.3.4. *In-silico* pharmacokinetic ADME predictions

**Figure 5:**
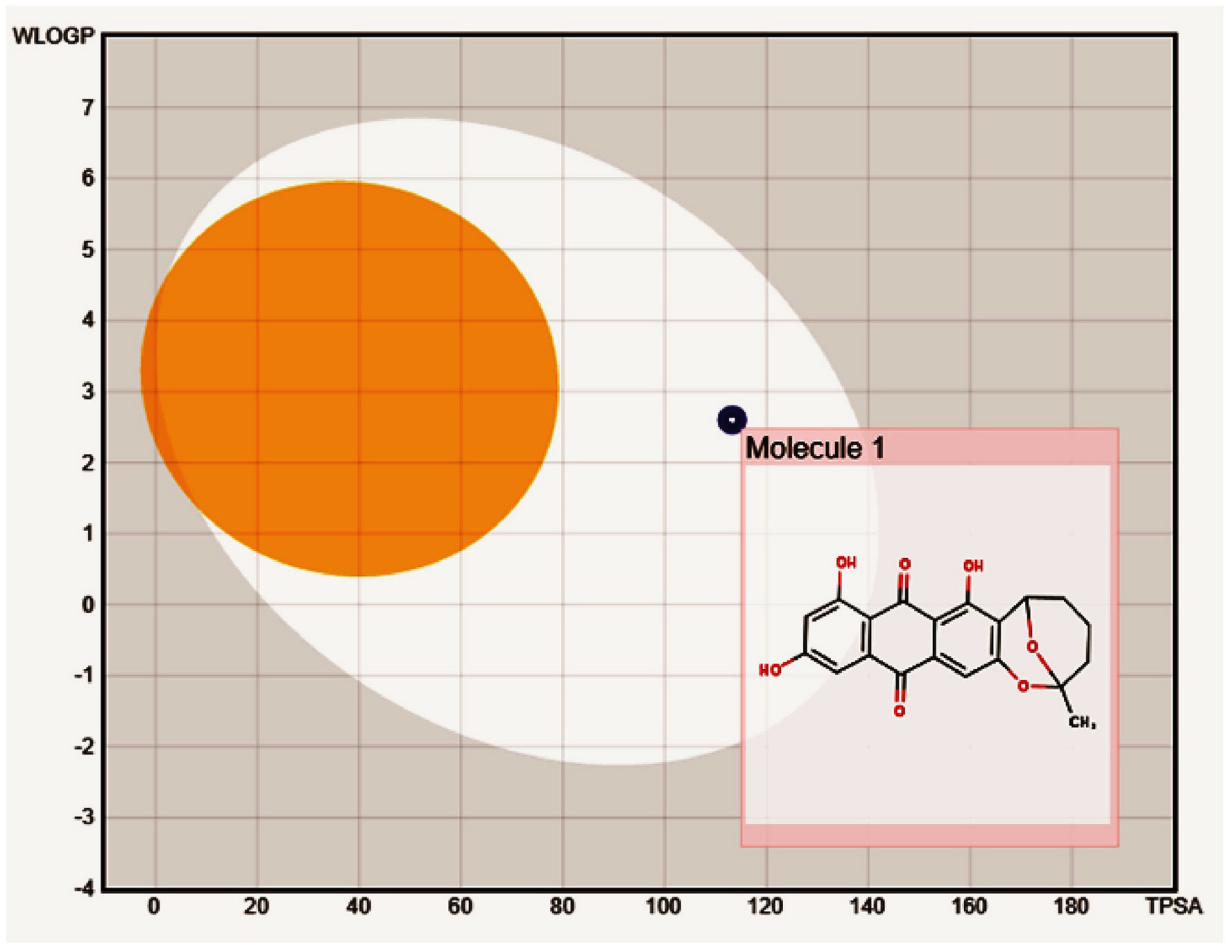
Boiled egg model to depict the gastrointestinal and blood-brain barrier solubility nature of compound averufin.

Pharmacokinetic ADME predictions of averufin revealed considerable results for plausible drug properties. As per Lipinski's rule of five compound averufin has three hydrogen bond donors and seven hydrogen bond acceptors. The molecular weight, log P-value, and molar refractivity are within the acceptable limit of 368.34 g/mol, 2.98, and 93.40, respectively (Table 3). The Osiris pharmacokinetic analysis (Table 4) revealed that compound (1) averufin has no mutagenic, tumorigenic, and reproductive toxicity, which strongly support the consideration of this compound for future pharmacological screening for *in-vivo* experiments as this compound has a good drug score. The boiled egg simulation shows that the compound averufin (1) is hydrophilic, which is a good hallmark of its palatability in gastrointestinal digestion (Figure 5). Above all, the oral bioavailability of averufin is marked as well, both by Veber's and Egan's rules. This analysis revealed that all the pharmacokinetic parameters of averufin are within the permissible range for human use, which strongly suggests that averufin could be a potential drug-like molecule.

**Table 3:** Lipinski rule-ADME validation for the compound averufin.

**Compound**	**Molecular weight (g/mol)**	**Lipophilicity (MLogP)**	**H-bond donors**	**H-bond acceptors**	**Rule violations**	**Drug-likeness**	**Status**
Averufin	368.34	2.98	3	7	1	Yes	Accepted

**Table 4:** Osiris pharmacokinetic rule for the compound averufin.

**Compound**	**Mutagenic**	**Tumorigenic**	**Irritant**	**Reproductive toxicity**	**Drug Score**	**Status**
Averufin	No	No	Slightly	No	0.15	Accepted

#### 3.3.5. Molecular dynamics study

**Figure 6:**
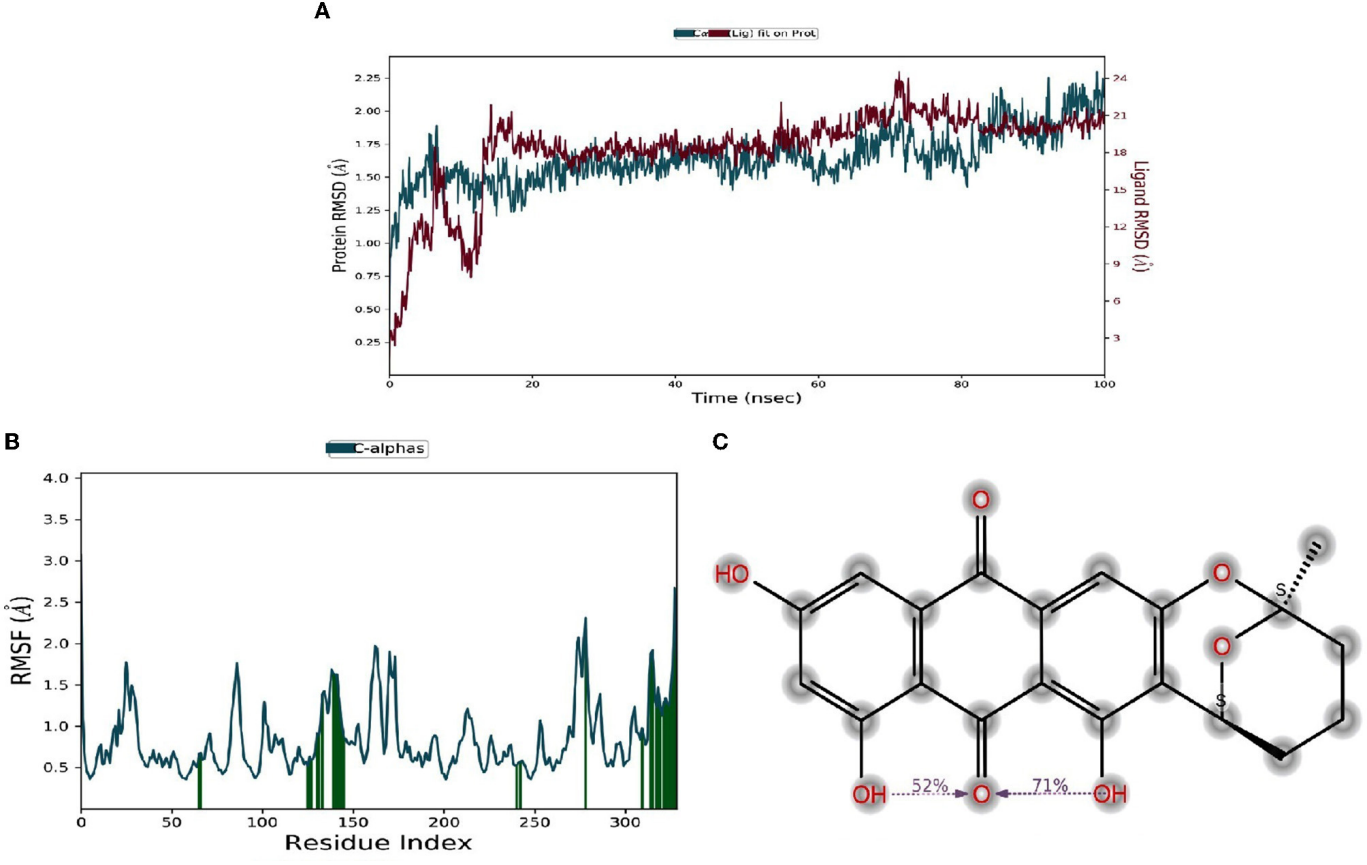
**(A–C)** Molecular dynamic studies of compound Averufin with HL-60 antigen 1A8E.

Molecular dynamic results revealed the ligand-protein interaction stability over time. The root means square deviation (RMSD) analysis report states that the compound (1) averufin binds with the target protein (1A8E). At the arrival time of simulation, it displayed a steady state of around 0.15 ns to the completion until 100 ns, which is a hallmark for good drug target interaction stability without drug binding displacement from the 1A8E protein target domain (Figure 6A). The root means square fluctuation (RMSF) prediction for human transferrin protein (1A8E) flexibility is depicted in Figure 6B; it reveals that peaks indicate areas of the protein that fluctuate the most during the simulation. Typically, the protein tails (N- and C-terminal) fluctuate more than any other part of the protein. Secondary structure elements, such as alpha helices and beta strands, are usually more rigid than the unstructured part of the proteins and thus fluctuate less than the loop regions. Protein residues that interact with the ligand are marked with green-colored vertical bars, showing that the ligand interacts with protein maximum at 130–145 ns time scale (Figure 6C). The protein-ligand interaction studies show that the maximum interaction of ligand averufin of 71% takes place through its –OH functional groups that binds the target proteins with asparagine and glutamine amino acids, which was further confirmed from Figure 4.

## 4. Discussion

Marine-derived natural products have seemingly attracted the interest of pharmacologists worldwide in recent years. Sponge-derived fungi account for the majority of the novel compounds (28%) reported from marine isolates of fungi (Hong et al., 2007; Hawas and El-Beih, 2012). Secondary metabolites derived from fungi are widely categorized as flavonoids, quinones, alkaloids, terpenoids, polyketides, isocoumarin derivatives, steroids, phenolic acids, and peptides with intriguing bioactive properties (Hajjaj et al., 2000; Ouyang, 2006; Zhang et al., 2009; Tian et al., 2015a,b). In the present study, seven different metabolites were isolated from the sponge-derived fungi *P.verruculosum* strain. Compounds (1-7) are of polyketide, quinone, and phenone derivatives. All seven isolated compounds were subjected to different biological assays, which include anticancer, antimicrobial, antiviral, and anti-tuberculosis screening. Compound (1) averufin exerts strong anticancer activity against HL60 (Human leukemic cell lines), with a strong IC_50_ value of 1.005μm concentration prominent at that of standard drug taxol. Reports have suggested that Averufin isolated from a marine-derived *Penicillium flavidorsum* SHK1-27 exerted a weak antitumor activity toward K562 cell lines with MIC values of 72.4 μmol/L (Hong et al., 2007). Compound (5), methyl emodin, exerts a weak anticancer effect against the tested cell lines. In a previous study, methyl-emodin tended to exert a moderate anticancer effect against the SGC-7901 cell line (Yang et al., 2009). Most of the compounds isolated herein have been reported from the *P.verruculosum* strain for the first time. Compound (2), a recently reported new compound from *Aspergillus versicolor* (Wu et al., 2016), is an interesting incidence and a first-time report from the *P.verruculosum* strain.

Molecular docking is a virtual technology that allows analyzing the precise drug-target interactions at the molecular level. With the advent of molecular docking studies, drug discovery and development have become convenient, especially for certain viral diseases like COVID-19 and Severe Acute Respiratory Syndrome (SARS) (Quimque et al., 2023). A plant-derived phenolic compound named Kobophenol-A binds with Spike protein receptors of both SARS-CoV-2 effectively, which were screened through virtual docking simulations, revealing that many natural products can be a potential drug target for many viral diseases (Gangadevi et al., 2021). Since compound (1) averufin exerts a strong anticancer effect on the HL60 cell line, the antigenic targets of HL60 cell lines 1AE, 1JFF, 2JOP, and 3BKY were docked with averufin and the standard drug taxol. ADME-Tox prediction helps assess the drug and non-drug properties with a high probability of success or failure based on the drug mimickers for a molecule. Averufin was recorded with good binding energy, and its pharmacokinetic ADME pharmacokinetic analysis of averufin revealed that it is oral bioavailability and drug score based on OSIRIS and Lipinski rule, which are good indicators of drug validation in prospect. *P.verruculosum*, an endophytic fungus, has been reported to produce intriguing secondary metabolites and polyketide compounds with prominent anticancer activity. Monascorubrine and monascin are the groups of polyketide compounds from *P. verruculosum* that show considerable cytotoxic activity toward KA3IT cancer cells (Shah et al., 2014). The dynamic molecular study of compound (1) averufin with target protein 1A8E confirms that the drug-target interaction is good without any displacement up to 100 ns. A previous study on sulfonamide-benzoxazoles, a synthetic chemical drug was docked with HL-60 antigens, revealed that the drug showed the same interaction with minimal saturation (Oksuzoglu et al., 2017). Understanding how polyketide structural variation is generated is key to identifying new products encoded in the vast number of emerging sequenced microbial genomes and developing new bioactive polyketides through rational pathways or enzyme engineering (Crawford et al., 2010). The results indicate that averufin, an aromatic polyketide, is a potent tumor inhibitor against HL60, a human myeloid leukemia cell line, and it can be beneficial in the therapy of leukemic treatment in the future. The present study also highlights that sponge endophyte *P. verruculosum* is a promising source of natural bioactive compounds.

## 5. Conclusions

In this study, we have evaluated the different metabolites isolated from sponge-derived fungi *P.verruculosum*. All of its structural characterization by NMR spectral studies speculate the chemical diversity of the fungus. Experimental results have shown the biomedical importance of isolated metabolites. Some of the isolated compounds have been reported from this fungus for the first time. The *in-vitro* and *in-silico* experimental findings of the compound averufin as a potent anticancer agent against leukemic HL-60 cell lines and its target antigens would also be helpful for researchers to conduct further *in-vitro* and *in-vivo* experimental studies for future applications.

## Data availability statement

The datasets presented in this study can be found in online repositories. The names of the repository/repositories and accession number(s) can be found in the article/Supplementary material.

## Author contributions

KK and LS equally contributed to the experimental work. YL and RK performed experimental supervision. SS and ZJ performed molecular docking studies, molecular dynamics, and data interpretations. SE and KS conducted manuscript drafting and reviewing. NMA, NA, and MS performed data interpretation. All authors reviewed and approved the final version of the manuscript.
