# Supplementary material for: Isolation of anticancer bioactive secondary metabolites from the sponge-derived endophytic fungi Penicillium sp. and in-silico computational docking approach
Source: Front Microbiol. 2023 Oct 2;14:1216928. doi: 10.3389/fmicb.2023.1216928 (PMC10577379; doi:10.3389/fmicb.2023.1216928)
Supplement: Supplementary file 2 [file Data_Sheet_1.PDF]

Supplementary materials

Isolation of anticancer bioactive secondary metabolites from the sponge-derived endophytic fungi *Penicillium* sp and In-silico computational docking approach.

Table of Contents

Fig. S1a ^{1}H NMR spectrum (500 MHz, DMSO) of compound **(1):** Averufin

Fig. S1b ^{13}C NMR spectrum (125 MHz, DMSO) of compound **(1):** Averufin

Fig. S2a ^{1}H NMR spectrum (500 MHz, DMSO) of compound **(2):** Aspergilol-A

Fig. S2b ^{13}C NMR spectrum (125 MHz, DMSO) of compound **(2):** Aspergilol-A

Fig. S3a ^{1}H NMR spectrum (500 MHz, DMSO) of compound **(3):** Sulochrin

Fig. S3b ^{13}C NMR spectrum (125 MHz, DMSO) of compound **(3):** Sulochrin

Fig. S4a ^{1}H NMR spectrum (500 MHz, DMSO) of compound **(4):** Monomethyl Sulochrin

Fig. S4b ^{13}C NMR spectrum (125 MHz, DMSO) of compound **(4):** Monomethyl Sulochrin

Fig. S5a ^{1}H NMR spectrum (500 MHz, $CDCL_3$) of compound **(5):** Methyl Emodin

Fig. S5b ^{13}C NMR spectrum (125 MHz, $CDCL_3$) of compound **(5):** Methyl Emodin

Fig. S6a ^{1}H NMR spectrum (500 MHz, $CDCL_3$) of compound **(6):** Citreoresin

Fig. S6b ^{13}C NMR spectrum (125 MHz, $CDCL_3$) of compound **(6):** Citreoresin

Fig. S7a ^{1}H NMR spectrum (500 MHz, $CDCL_3$) of compound **(7):** Diorcinol

Fig. S7b ^{13}C NMR spectrum (125 MHz, $CDCL_3$) of compound **(7):** Dirocinol

Table S1. Anticancer activity of compound 1–10 (MIC, μM)

Table S2. Antitubercular activity of compound 1–10 (MIC, μM)

Table S3. Antiviral and Antimicrobial activity of compound 1–10 (MIC, nM & μM)

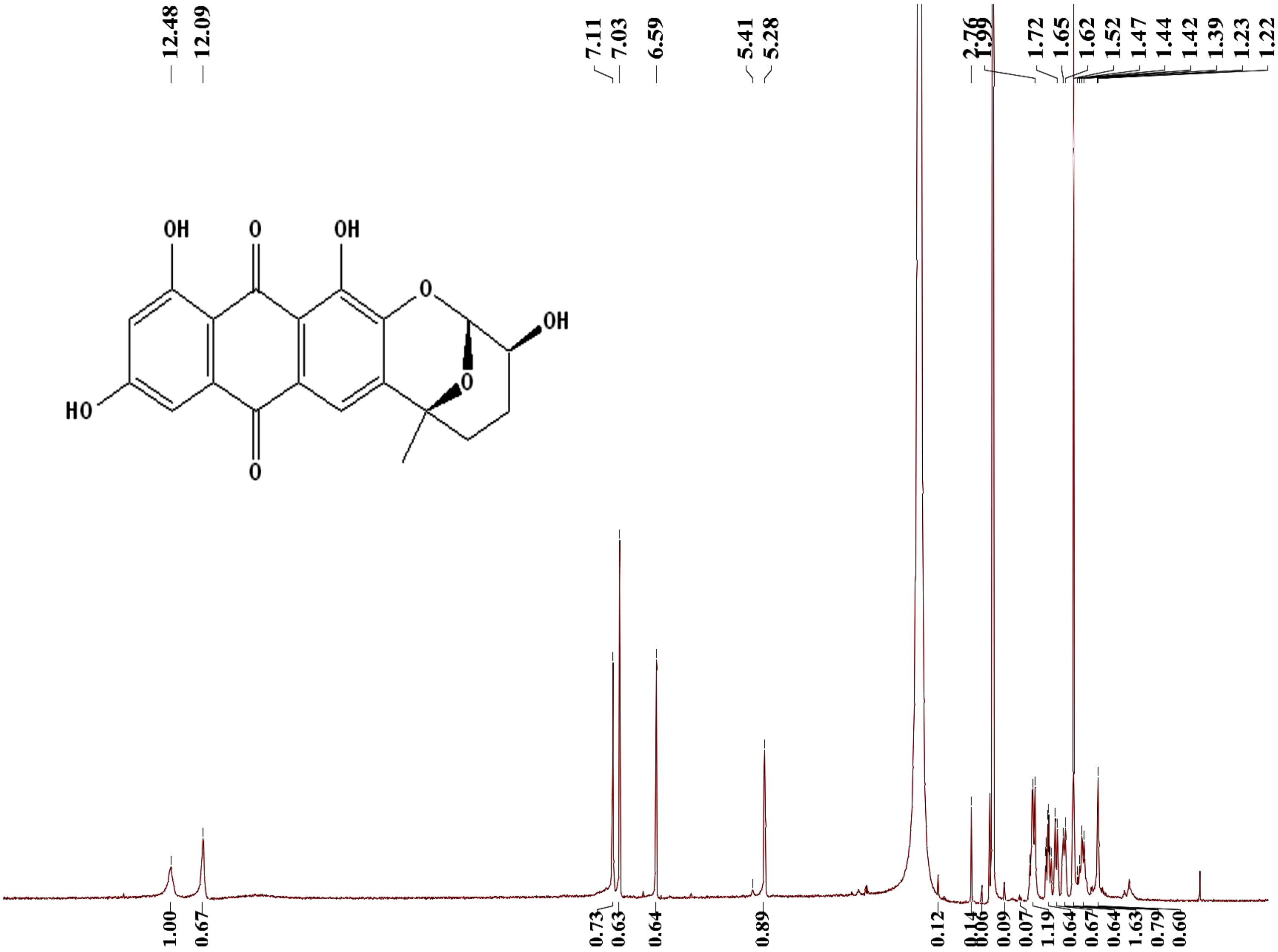

Fig. S1a: ^{1}H NMR spectrum (500 MHz, DMSO) of compound **(1):** Averufin

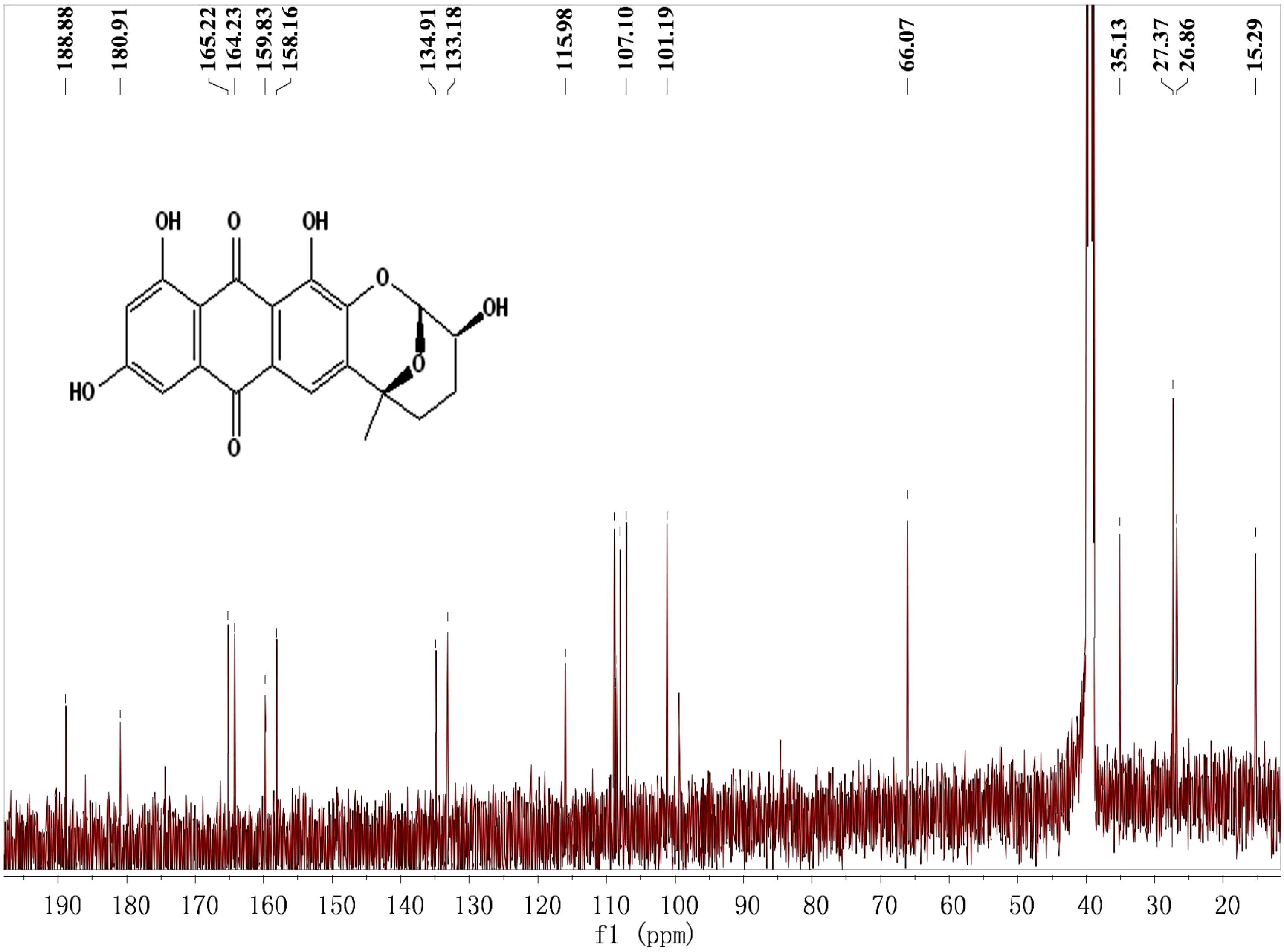

Fig. S1b: ^{13}C NMR spectrum (125 MHz, DMSO) of compound **(1):** Averufin

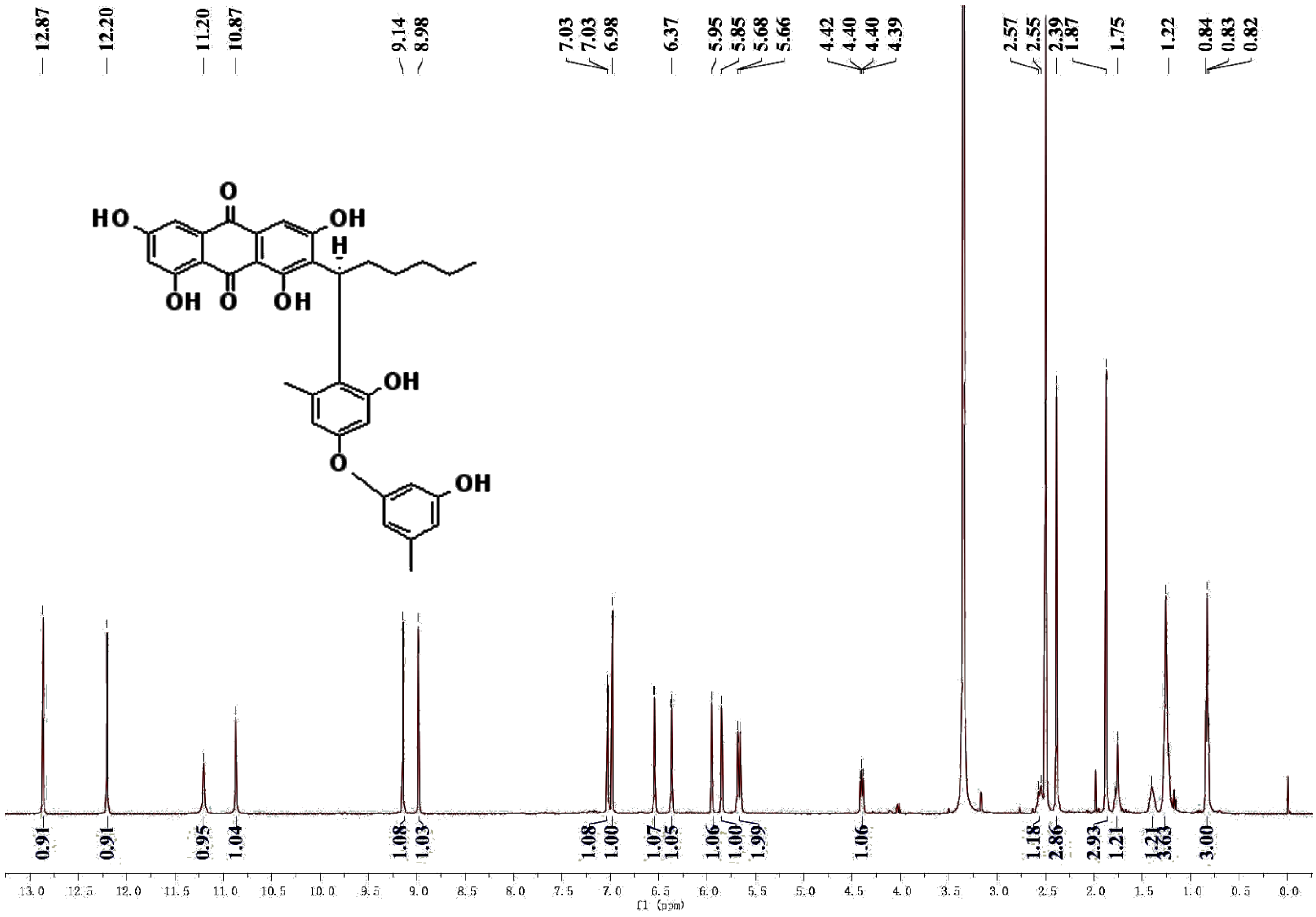

Fig. S2a: ^{1}H NMR spectrum (500 MHz, DMSO) of compound **(2):** Aspergilol-A

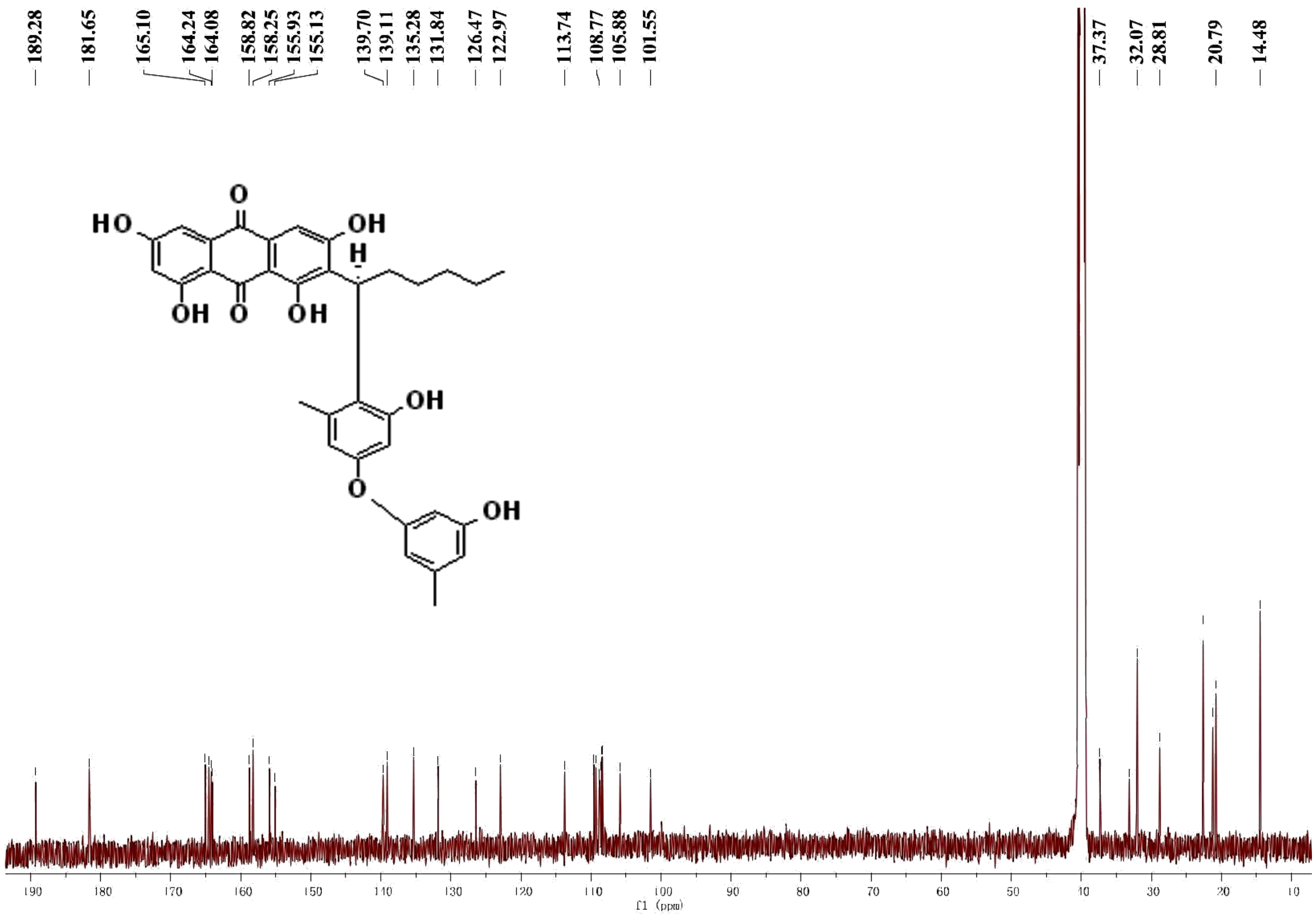

Fig. S2b: ^{13}C NMR spectrum (125 MHz, DMSO) of compound **(2):** Aspergilol-A

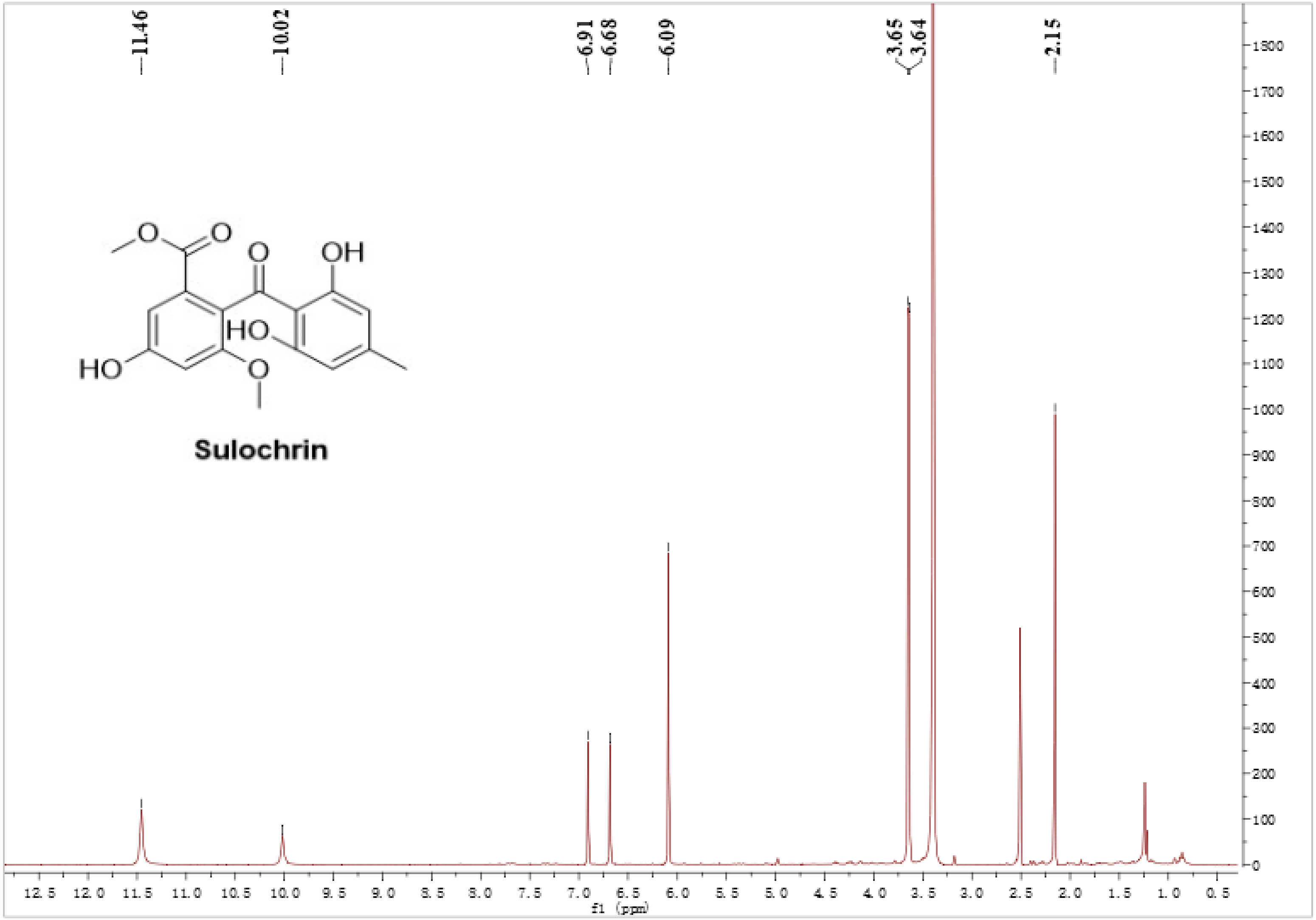

Fig. S3a ^{1}H NMR spectrum (500 MHz, DMSO) of compound **(3):** Sulochrin

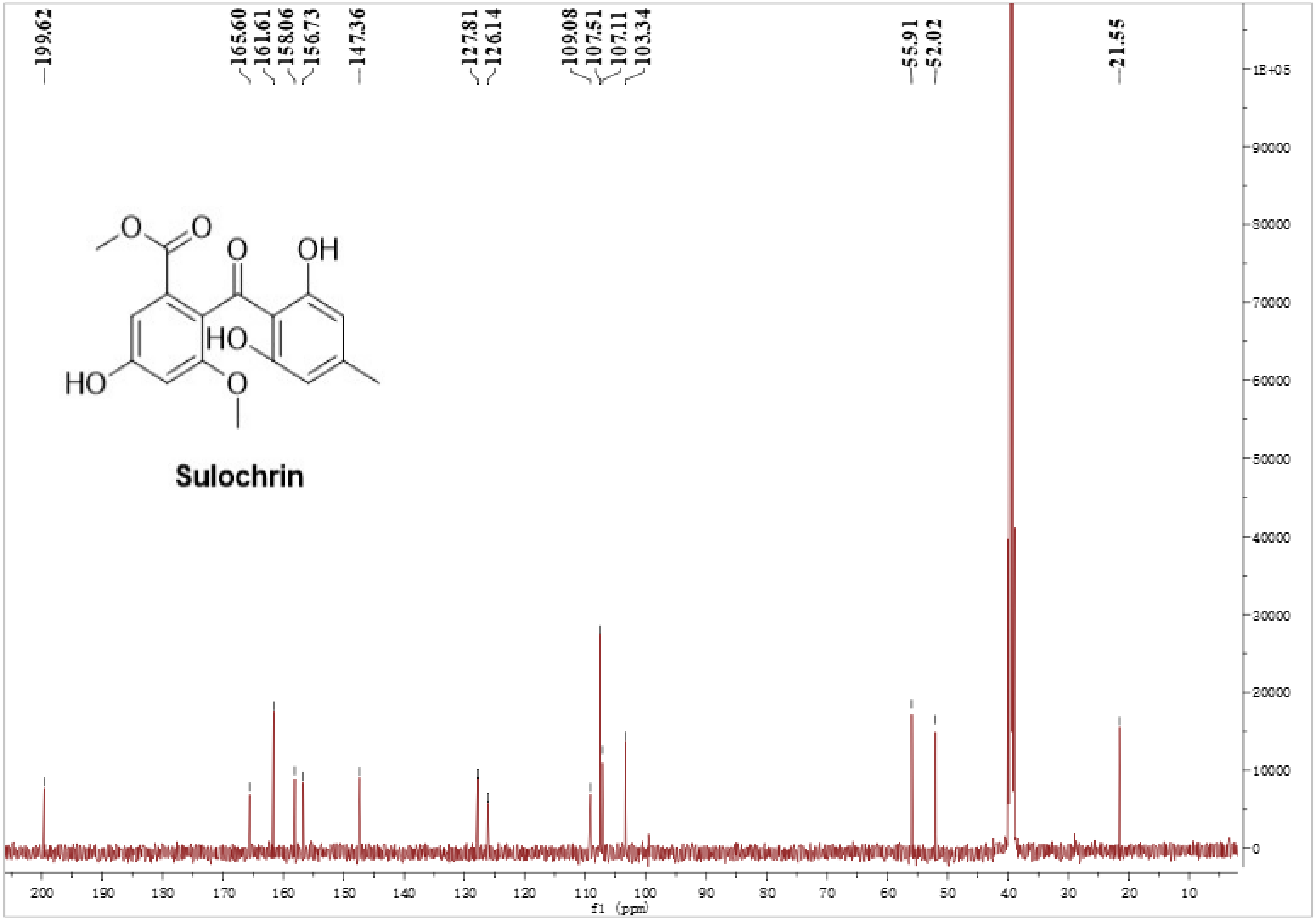

Fig. S3b: ^{13}C NMR spectrum (125 MHz, DMSO) of compound **(3):** Sulochrin

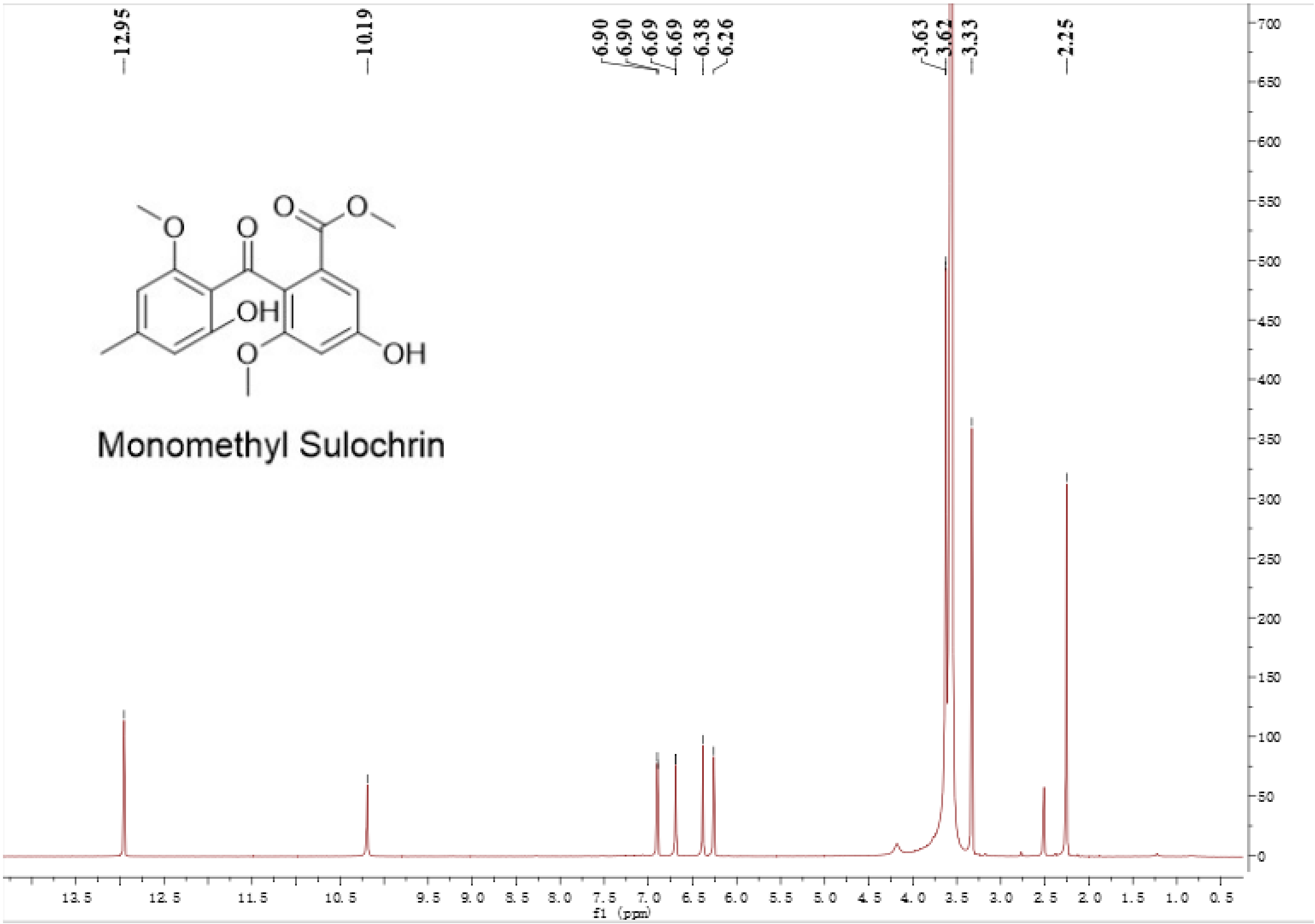

Fig. S4a ^{1}H NMR spectrum (500 MHz, DMSO) of compound **(4):** Monomethyl Sulochrin

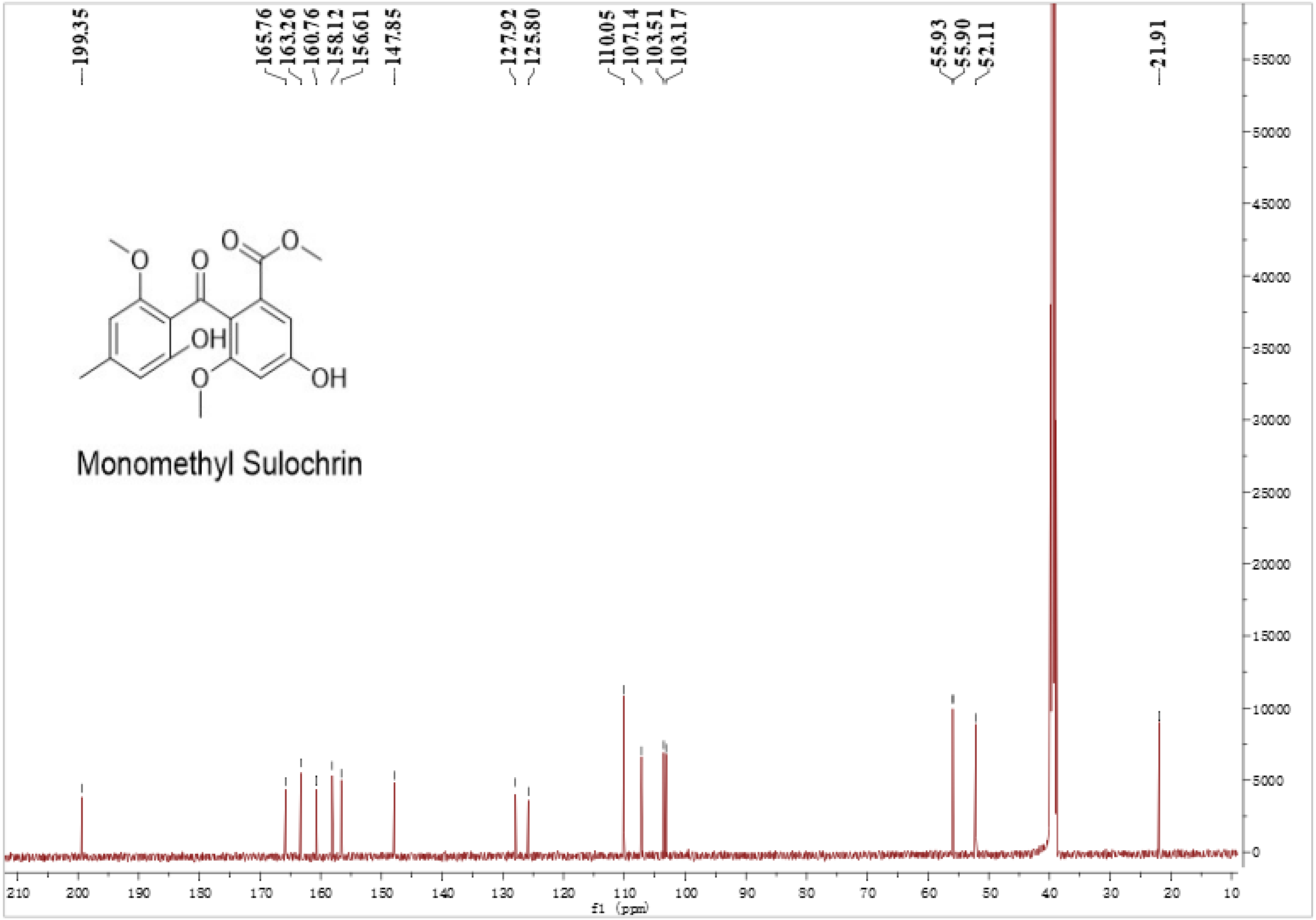

Fig. S4b ^{13}C NMR spectrum (125 MHz, DMSO) of compound **(4):** Monomethyl Sulochrin

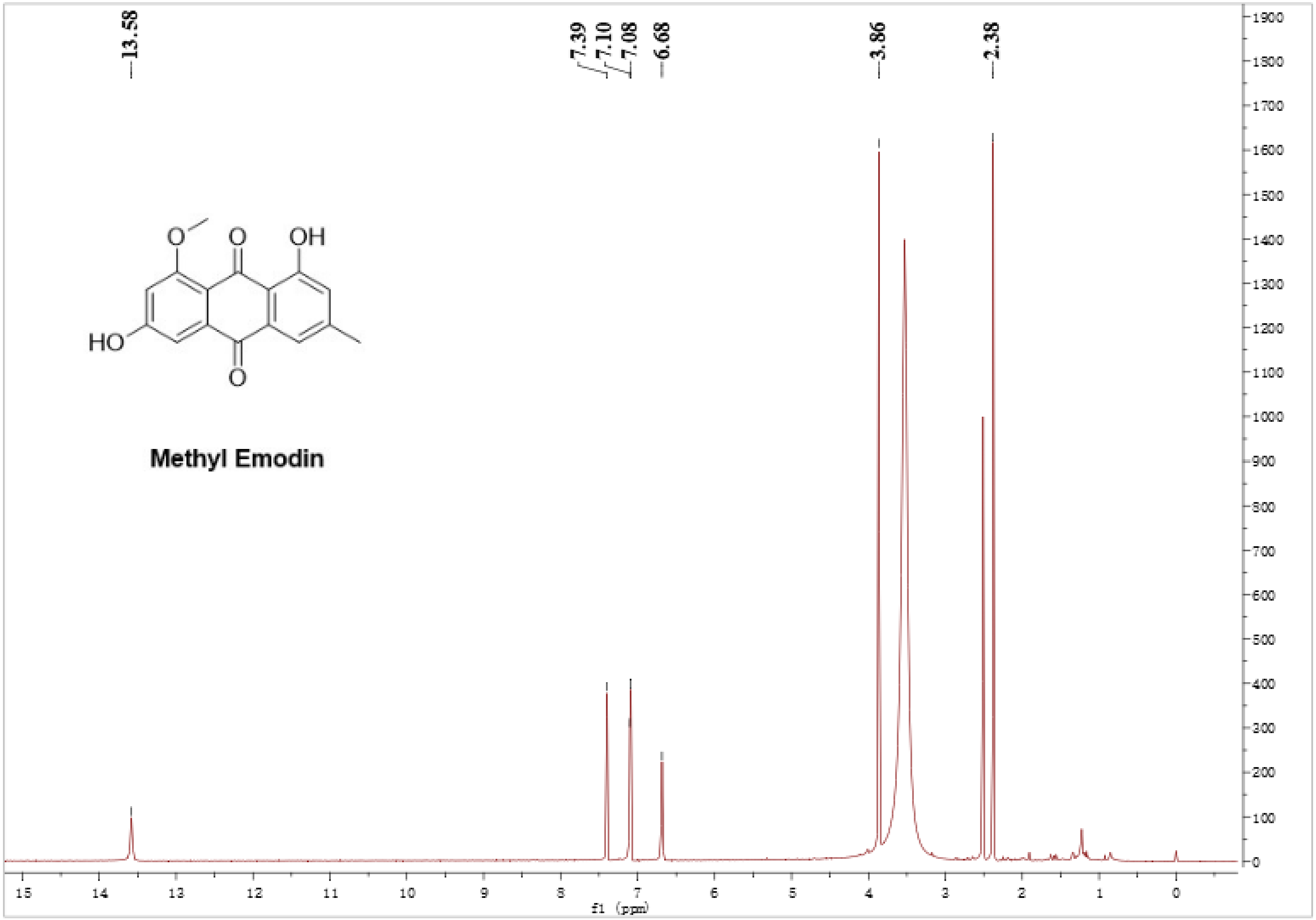

Fig. S5a ^{1}H NMR spectrum (500 MHz, DMSO) of compound **(4):** Methyl Emodin

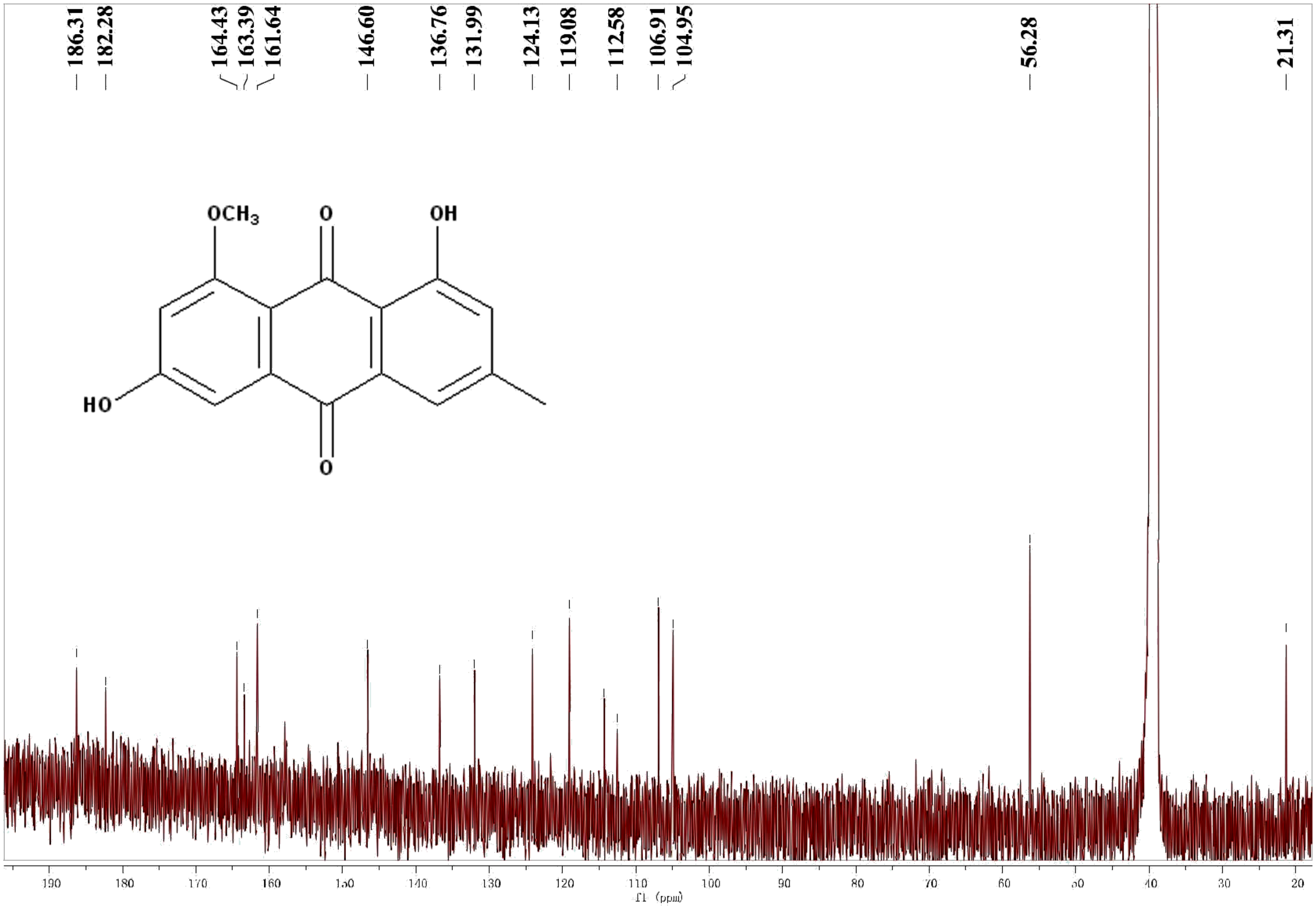

Fig. S5b ^{13}C NMR spectrum (125 MHz, DMSO) of compound **(4):** Methyl Emodin

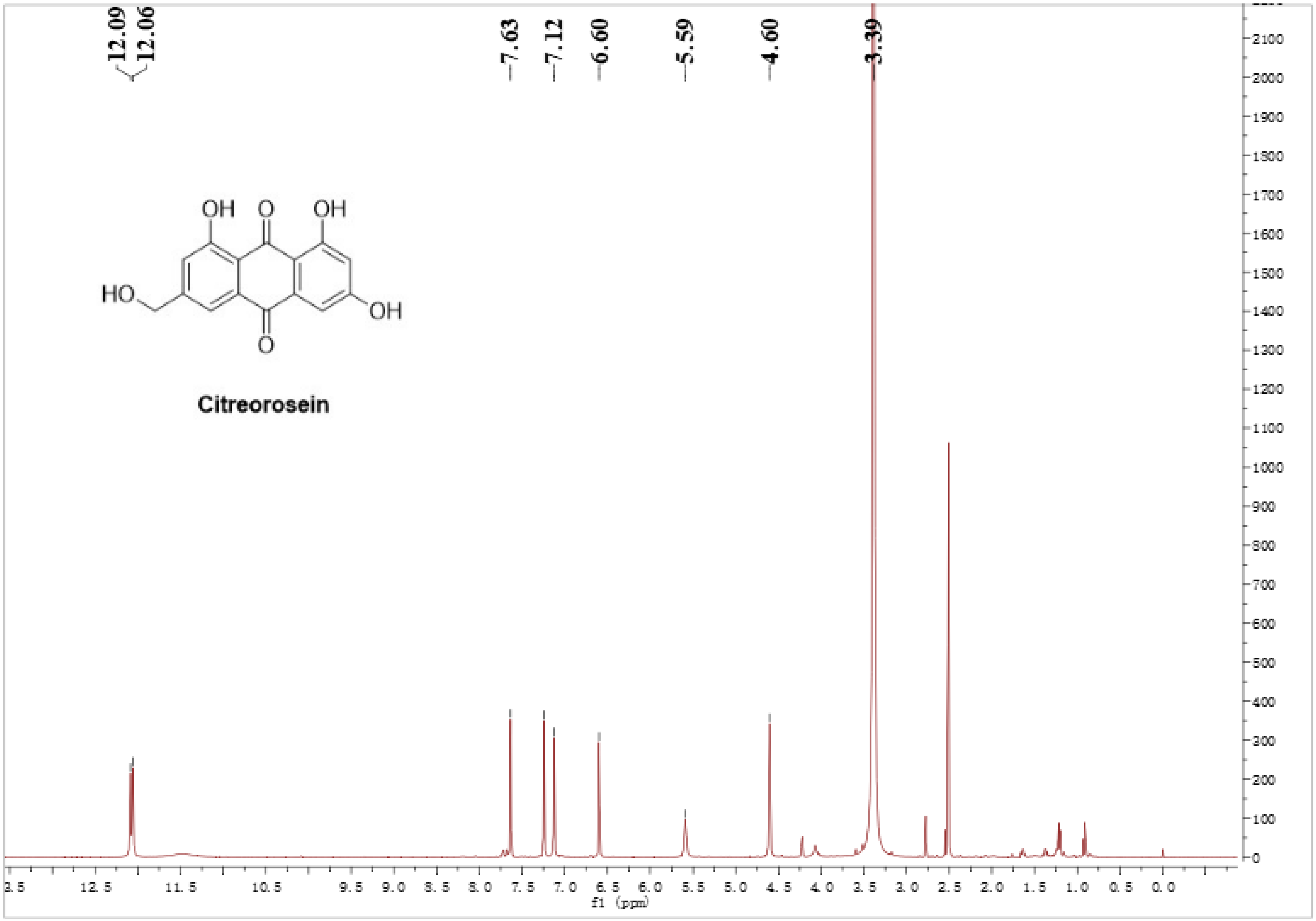

Fig. S6a ^{1}H NMR spectrum (500 MHz, CDCL$_3$) of compound **(6):** Citreoresin

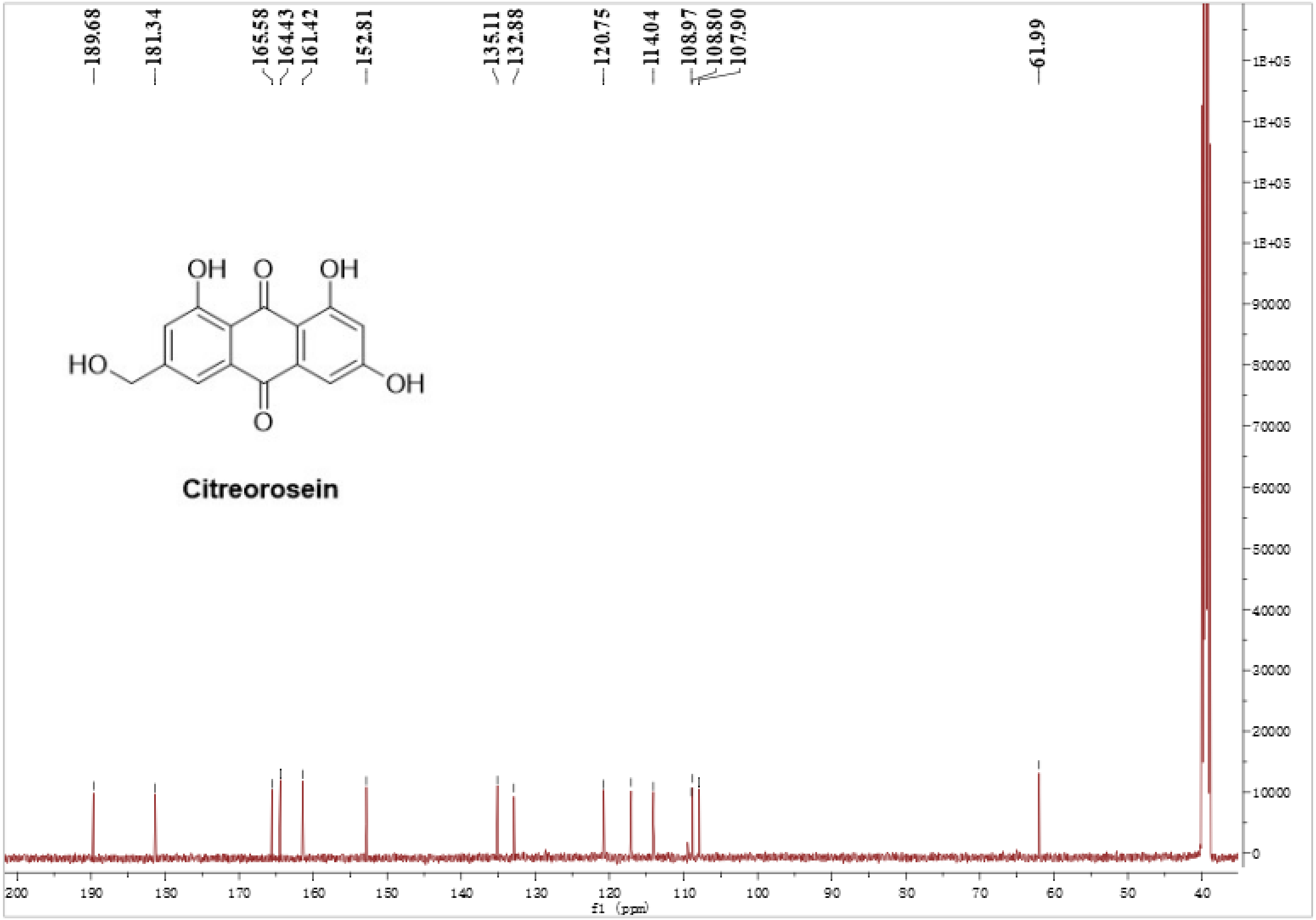

Fig. S6b –C NMR spectrum (125 MHz, $CDCL_3$) of compound **(6):** Citreoresin

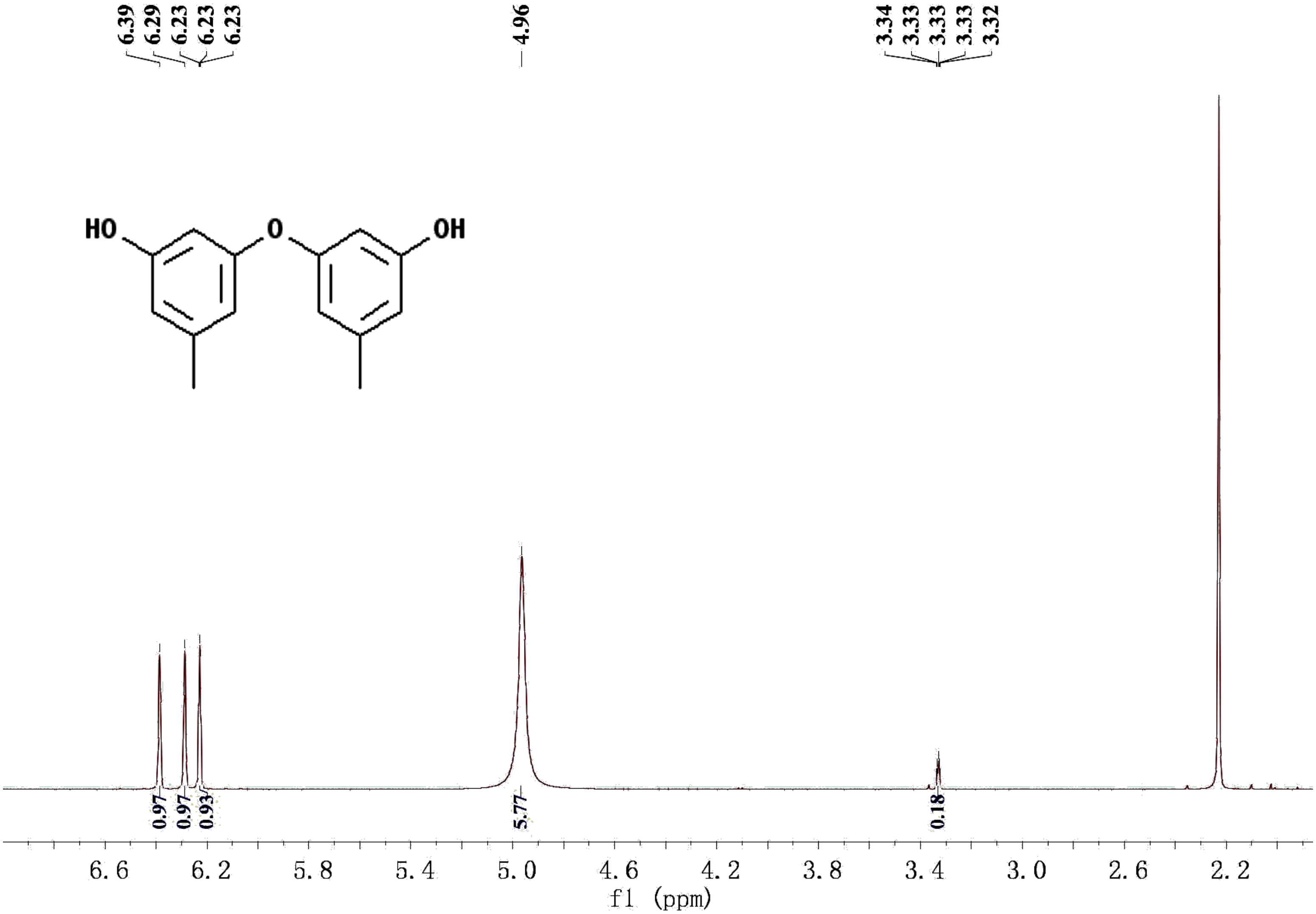

Fig. S7a ^{1}H NMR spectrum (500 MHz, CD_3OD) of compound **(9):** Diorcinol

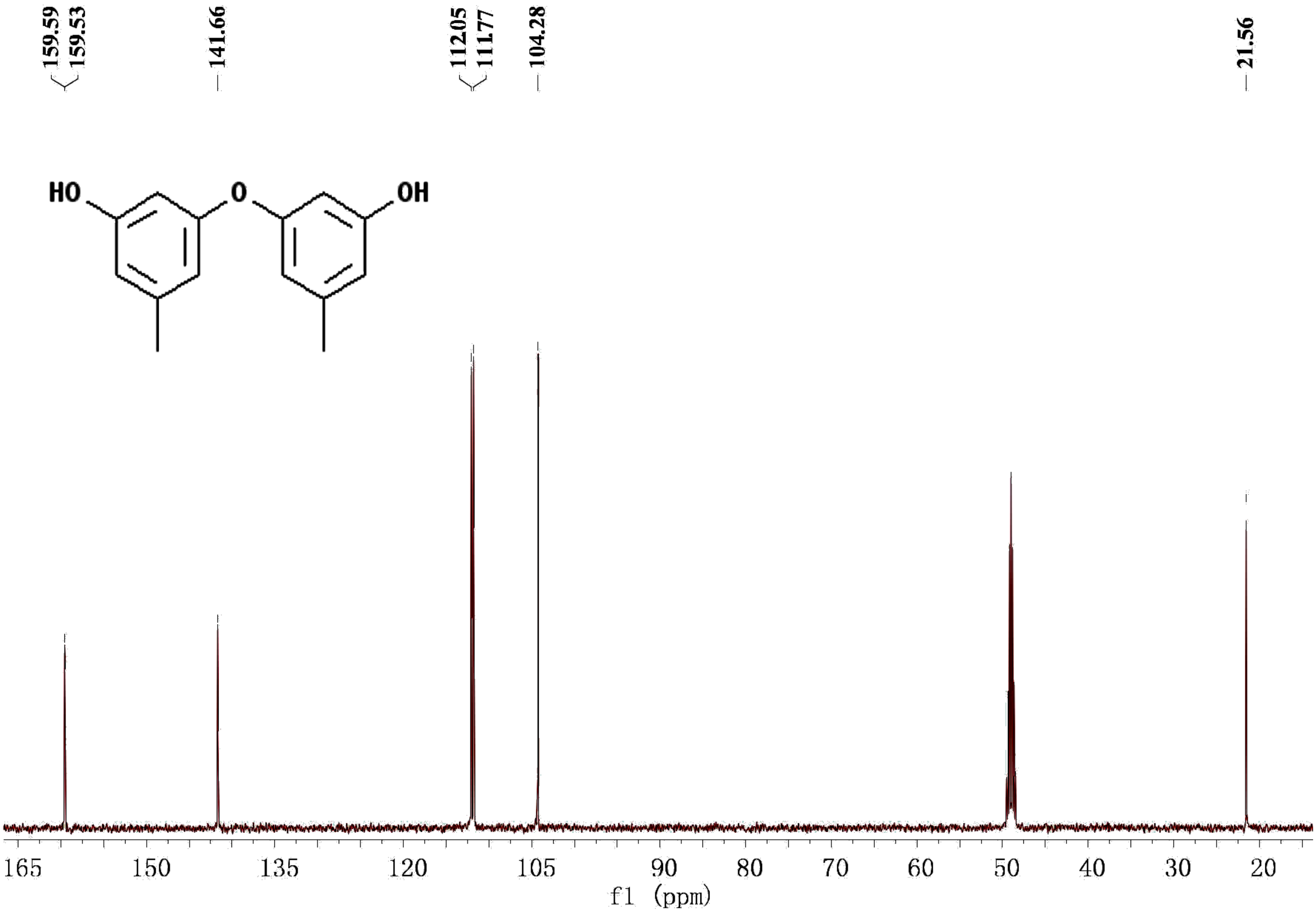

Fig. S7b ^{13}C NMR spectrum (125 MHz, CD_3OD) of compound **(9):** Diorcinol

Table S1. Anticancer activity of compound 1–7 (MIC, µM)

Cpd	K562	A549	DU145	H1975	MCF-7	Huh-7	HL7702	HL60	HeLa	MOLT-4	MCF-10A
**1**	17.4±0.01	76.1±0.01	91.2±0.01	8.64±0.01	6.71±0.01	3.13±0.01	3.57±0.01	1.005±0.01*	8.11±0.01	5.77±0.01	≥50
**5**	16.3±0.01	81.3±0.01	101.4±0.01	43.5±0.01	54.3±0.01	25.1±0.01	67.2±0.01	13.2±0.01	24.9±0.01	13.6±0.01	≥50
**Taxol**	0.003±0.01	0.024±0.01	0.015±0.01	0.014±0.01	0.002±0.01*	0.003±0.01	0.003±0.01	0.002±0.01*	0.003±0.01	0.003±0.01	≥50
**SI**	1.22	1.87	3.11	1.24	1.78	2.47	3.89	13.47	3.24	2.47	69.41

Data are computed as standard deviation mean ± SD, n = 3, SI (selective index), $*p \leq 0.05$ (one-way ANOVA).

Table S2. Antitubercular activity of compound 1–7 (MIC, µM)

Compound	Antitubercular activity
**1**	>50
**2**	>50
**3**	>50
**4**	>50
**5**	>50
**6**	>50
**7**	>50
**INH**	3.98

*INH - Isoniazid

Table S3. Antiviral and Antimicrobial activity of compound 1–7 (MIC, nM & µM)

**Compound**	**Antiviral (IC_{50} nM)**	
	**H_1N_1**	**H_3N_2**
**1**	>50	>50
**2**	>50	>50
**3**	>50	>50
**4**	>50	>50
**5**	>50	>50
**6**	>50	>50
**7**	>50	>50
**Tamiflu**	15.2	17.6

NA-No Activity
